# Structural insights on the efficient catalysis of hydroperoxide reduction by Ohr: Crystallographic and molecular dynamics approaches

**DOI:** 10.1371/journal.pone.0196918

**Published:** 2018-05-21

**Authors:** Erika Piccirillo, Thiago G. P. Alegria, Karen F. Discola, José R. R. Cussiol, Renato M. Domingos, Marcos A. de Oliveira, Leandro de Rezende, Luis E. S. Netto, Antonia T-do Amaral

**Affiliations:** 1 Departamento de Química Fundamental, Instituto de Química, Universidade de São Paulo, São Paulo, SP, Brazil; 2 Departamento de Genética e Biologia Evolutiva, Instituto de Biociências, Universidade de São Paulo, São Paulo, SP, Brazil; 3 Instituto de Biociências, Campus do Litoral Paulista, Universidade Estadual Paulista Júlio de Mesquita Filho, São Vicente, SP, Brazil; University of Queensland, AUSTRALIA

## Abstract

Organic hydroperoxide resistance (Ohr) enzymes are highly efficient Cys-based peroxidases that play central roles in bacterial response to fatty acid hydroperoxides and peroxynitrite, two oxidants that are generated during host-pathogen interactions. In the active site of Ohr proteins, the conserved Arg (Arg19 in Ohr from *Xylella fastidiosa*) and Glu (Glu51 in Ohr from *Xylella fastidiosa*) residues, among other factors, are involved in the extremely high reactivity of the peroxidatic Cys (C_p_) toward hydroperoxides. In the closed state, the thiolate of C_p_ is in close proximity to the guanidinium group of Arg19. Ohr enzymes can also assume an open state, where the loop containing the catalytic Arg is far away from C_p_ and Glu51. Here, we aimed to gain insights into the putative structural switches of the Ohr catalytic cycle. First, we describe the crystal structure of Ohr from *Xylella fastidiosa* (XfOhr) in the open state that, together with the previously described XfOhr structure in the closed state, may represent two snapshots along the coordinate of the enzyme-catalyzed reaction. These two structures were used for the experimental validation of molecular dynamics (MD) simulations. MD simulations employing distinct protonation states and *in silico* mutagenesis indicated that the polar interactions of Arg19 with Glu51 and C_p_ contributed to the stabilization of XfOhr in the closed state. Indeed, C_p_ oxidation to the disulfide state facilitated the switching of the Arg19 loop from the closed to the open state. In addition to the Arg19 loop, other portions of XfOhr displayed high mobility, such as a loop rich in Gly residues. In summary, we obtained a high correlation between crystallographic data, MD simulations and biochemical/enzymatic assays. The dynamics of the Ohr enzymes are unique among the Cys-based peroxidases, in which the active site Arg undergoes structural switches throughout the catalytic cycle, while C_p_ remains relatively static.

## Introduction

Oxidants such as fatty acid hydroperoxides are signaling molecules involved in host-pathogen interactions, and therefore, their levels are strictly controlled by peroxidases and other mechanisms [1–4]. Ohr (Organic hydroperoxide resistance) proteins are Cys-based, dithiol-dependent peroxidases that display unique biochemical and structural properties [5,6]. Ohr enzymes play central roles in the bacterial response to peroxynitrite and fatty acid hydroperoxides, two oxidants involved in host–pathogen interactions [1]. These enzymes are found in bacteria and fungi, and they are absent in their hosts (plants and animals) [7], making them promising targets for drug discovery. Some examples of pathogenic bacteria that express Ohr proteins are *Pseudomonas aeruginosa*, *Vibrio cholerae* and *Xyllela fastidiosa* [7]. *Xylella fastidiosa* is a plant pathogen with agronomic interest, causing disease in citrus, grapes and olives [8].

Ohr protein was first identified in *Xanthomonas campestris* pv. *phaseoli* due to its involvement in the bacterial response to organic hydroperoxides, but it is not involved in the H_2_O_2_ response [9]. This unusual organic hydroperoxide resistance phenotype is related to the ability of Ohr enzymes to reduce organic hydroperoxides with higher efficiency than H_2_O_2_ [5,6,10]. Ohr, Prxs (peroxiredoxins) and Gpx (GSH peroxidases) are all Cys-based, thiol-dependent peroxidases; however, Ohr and Prx/Gpx enzymes belong to distinct families, as their biochemical/enzymatic properties and structures are distinct [6, 11]. Instead, Ohr proteins share structural and amino acid sequence similarities with OsmC proteins, which were initially related to the bacterial response to osmotic stress [12]. Later, it was demonstrated that OsmC enzymes are also endowed with thiol peroxidase activity [13,14]. Therefore, Ohr/OsmC is a family of Cys-based proteins that also comprise proteins (such as YhfA from *Escherichia coli*) whose biochemical activity is still unknown [7,12,14].

Proteins belonging to the Ohr/OsmC family display a barrel-like structure formed by a tightly folded homodimer, in which two six-stranded β-sheets wrap around two central α-helices [6,11,15]. The two active sites are located at the dimer interface on opposite sides of the protein, and the reactive Cys, also called the peroxidatic Cys (C_p_, Cys61 in Ohr from *Xylella fastidiosa*—XfOhr), is located in one of the central α-helices. C_p_ and two other residues (Arg19 and Glu51 in XfOhr) constitute the catalytic triad. The involvement of catalytic Arg in the ability of Ohr enzymes to reduce hydroperoxides was directly assessed by site-directed mutagenesis in Ohr from *Pseudomonas aeruginosa* (PaOhr) [6]. The carboxylic group of catalytic Glu orients the guanidinium group of Arg toward C_p_ in a configuration that appears to be optimal for the reduction of organic hydroperoxides [6,11]. Recently, we showed that fatty acid hydroperoxides are biological substrates of Ohr enzymes [1], displaying properties expected for ligands of these enzymes, such as an elongated shape and hydrophobicity. Peroxynitrite is also one of the biological oxidants of Ohr enzymes, but other features are associated with this catalysis [1]. In spite of all these advances, several aspects related to the extremely high efficiency of Ohr enzymes to reduce hydroperoxides remains elusive, such as the possible occurrence of structural movements along the catalytic cycle.

The reaction of hydroperoxides with C_p_ generates a sulfenic acid (C_p_-SOH), which undergoes condensation with the resolving Cys (C_r_, which is Cys125 in XfOhr), generating an intramolecular disulfide bond [6,11]. Moreover, the loop that contains the catalytic Arg (herein named the Arg19 loop) was observed far away from C_p_ and the catalytic Glu in the crystal structure of Ohr from *Deinococcus radiodurans* (DrOhr) [15]. In this case, the two Cys residues form a disulfide bond [15]. Therefore, we previously hypothesized that Ohr enzymes in the so-called “closed state” [6,11] would present catalytic Arg in an orientation able to activate C_p_ for hydroperoxide reduction, whereas Ohr enzymes in the so-called “open configuration” [15] would be more prone to recycling by the reducing substrate. We have since shown that the reducing substrates of XfOhr are lipoylated proteins [10], in contrast to the Prx/Gpx counterparts that are mainly reduced by thioredoxin or GSH [16]. Here, for the first time, we present crystal structures for the same Ohr protein in the open and closed states, allowing for the validation of the *in silico* simulations. Additionally, to better understand the structural changes during the catalytic cycle, molecular dynamics (MD) simulations were applied to the XfOhr structure in its closed and open states, in distinct protonation and oxidation states, and after *in silico* mutagenesis. The same mutagenesis was also performed in the recombinant Ohr protein to evaluate its biochemical properties. Among other findings, our results indicate that polar interactions among the C_p_, Arg19 and Glu51 residues are important to stabilize XfOhr in the closed state, and they are also required to activate the thiolate for hydroperoxide reduction. The disruption of any of these polar interactions releases some of the constraints on the Arg19 loop movement.

## Materials and methods

### Crystallization trials, data collection and processing

The procedures concerning XfOhr expression and purification have been previously reported [5]. XfOhr (10 mg/ml) was treated with 1.2 mM lipoamide at 310 K for 1 h and crystallized using the hanging-drop vapor diffusion method. The optimal crystallization condition was obtained using reservoir solution pH 6.0 (0.1 M sodium cacodylate and 0.4 M sodium citrate). The XfOhr crystal, cryoprotected by the mother liquor solution supplemented with 20% glycerol, was cooled to 100 K in a nitrogen gas stream, and X-ray diffraction data were collected at protein crystallography beam line D03B-MX1 at the Brazilian Synchrotron Light Laboratory, LNLS. The data set was processed using the programs MOSFLM [17] and SCALA [18,19] from the CCP4i package [20].

### Structure determination, model building and refinement

The Matthews coefficient (2.18) revealed three Ohr chains per asymmetric unit, and the monomer structure of the XfOhr (1ZB8) was used as a search model in molecular replacement protocols using the program Phaser [21]. The model was constructed by consecutive cycles of manual modelling, using the program Coot [22], and refinement using Refmac [23]. The stereochemical parameters of the final model were evaluated using the programs PROCHECK [24] and WHATCHECK [25]. Cα superposition was performed using Coot [22], and molecular graphical representations were generated using PyMOL [26].

### Site-directed mutagenesis

The pET15b/XfOhr plasmid was used as a template to generate the individual Ohr mutants carrying mutations of Arg19 to Ala (R19A) and Glu51 to Ala (E51A). The mutagenesis protocols were performed according to the manufacturer’s instructions (Quick Change II Kit; Stratagene) with the following primers: XfOhrR19A_F (5’ CAACTGGTGGCGCCGATGGCAGC 3’), XfOhrR19A_R (5’ GCTGCCATCGGCGCCACCAGTTG 3’), XfOhrE51A_F (5’ GGTACCAATCCAGCGCAACTGTTTG 3’) XfOhrE51A_R (5’ CAAACAGTTGCGCTGGATTGGTACC 3’). The reaction products were treated with Dpn I to remove the parental methylated plasmids, and the *E*. *coli* XL1-Blue strain was used as the host and transformed by electroporation. Single colonies were selected and their plasmids were extracted and sequenced with the BigDye Terminator v3.1 Cycle Sequencing Kit using an automatic sequencer, the ABI 3730 DNA Analyzer (Thermo Scientific), to confirm the codon substitutions. The plasmids harboring the mutations were transformed into the *E*. *coli* BL21 (*DE3*) strain by electroporation. The procedures concerning XfOhr mutants expression and purification were the same as for the wild-type.

### Lipoamide-lipoamide dehydrogenase peroxidase-coupled assay

The lipoyl peroxidase activity levels of wild-type XfOhr and its mutants (R19A and E51A) were determined as previously described [10]. The reactions were followed by the decay of absorbance at 340 nm (e = 6,290 M^−1^·cm^−1^) due to NADH oxidation.

### pK_a_ determination by monobromobimane alkylation assay

Wild-type XfOhr and its mutants (R19A and E51A) were reduced with 100 mM DTT (dithiothreitol) for 2 hours at room temperature. The DTT excess was then removed by gel filtration (PD-10 desalting column—GE), and the Ohr proteins (10 μM) were incubated with monobromobimane (2 μM) in buffers (50 mM) at different pH values (3.0 to 7.0) for 20 minutes at room temperature. The rates of alkylation by monobromobimane were determined by extrapolation of the maximum inclination of the curves [27]. Subsequently, the pK_a_ values were determined by the Henderson-Hasselbach equation in GraphPad^®^Prism4.

### Circular dichroism

All measurements were carried out in Tris buffer (10 mM) pH 7.4, and wild-type XfOhr and its mutants (R19A and E51A) were used at 15 μM. CD spectra were recorded from 180 to 320 nm using a JASCO spectropolarimeter, model J720 at the Central Analítica of IQUSP, SP.

### Morph conformations

Morph conformations were generated using UCSF Chimera [28]. For this purpose, we applied the corkscrew interpolation method with 40 interpolation steps and used two crystal structures of XfOhr in its closed (1ZB8) and open states (4XX2) to generate the first set of morph conformations. Subsequently, the first, the average and the last snapshots of the XfOhr-SS trajectory (see below) were used to generate the second set of morph conformations.

### MD simulations

The XfOhr structure in the closed conformation (PDB entry 1ZB8, 2.4 Å resolution) was subjected to MD simulation studies in two conditions: (1) in the reduced form (C_p_ as thiolate, Cys61) with 12 crystal water molecules, having B-factors < 25 Å^2^ and (2) with an artificial intramolecular disulfide between C_p_ and Cys125, which was built with SYBYL [29]. After the disulfide bond formation, the neighboring residues had their geometry optimized using Tripos force field and the Powell method [30,31]. The XfOhr trajectories in the reduced and oxidized forms were named XfOhr-S^-^ and XfOhr-SS, respectively. Furthermore, an XfOhr trajectory with C_p_ as a protonated thiol (named XfOhr-SH) was similarly built and subjected to MD simulation. To evaluate the roles of the Arg19 and Glu51 residues in the conformational change of XfOhr, the R19A and E51A XfOhr mutants were built *in silico* using SYBYL-X [32]. The wild-type residues were replaced by alanine, and their neighboring residues were minimized, as described above.

Finally, the open conformation of XfOhr described here (PDB entry 4XX2) was also used as a starting point for MD simulation, having, however, its C_p_ reduced to thiolate by breaking the disulfide bond and deprotonated using SYBYL-X [32]. Subsequently, the geometry of the C_p_ neighboring residues was optimized as described for XfOhr-SS, and the minimized structure was named Open-S^-^. Arginine and lysine were protonated, whereas aspartic and glutamic acids were deprotonated. Histidine was protonated at its ε-nitrogen atoms.

All MD simulations were performed using GROMACS 4.6.3 [33,34] and G54a7 force field [35]. Force field parameters for cysteine as thiolate were taken from those available for Cys without adding a hydrogen atom to the Sγ atom. Partial charges for Sγ and Cβ atoms were assigned as -0.7 and -0.3, respectively, which correspond to the mean values calculated using the Gasteiger Marsili, Hückel, Pullman, MMFF94, Gasteiger Hückel methods available in SYBYL-X [32]. The starting structure was initially minimized in vacuum, using the steepest descent method and the conjugated gradient algorithm (2000 steps each). The minimized structure was placed in a 100 Å cubic box, solvated with simple point-charge (SPC) water [36] and neutralized by adding sodium ions. Periodic boundary conditions were applied, and all covalent bonds containing hydrogen were fixed at equilibrium lengths using the LINCS algorithm [37]. The particle-mesh Ewald method [38,39] was used and a 9 Å cutoff value was applied for van der Waals interactions. The system energy was further minimized using the steepest descent method and the conjugate gradient method (2000 steps each). Subsequently, a position restraint dynamics simulation was performed for 2.5 ps at 200 K, keeping rigid all protein atom positions. The whole system was heated from 100 K to 300 K over 37 ps, followed by a period of 100 ps of equilibration. The temperature and pressure were kept at 300 K and 1 atm, respectively, by the V-rescale [40] and Berendsen [41] approaches. Subsequently, MD simulations were carried out for 50/150 ns at 300 K. A 2 fs integration time step was used, and configurations were collected every 2 ps.

VMD [42] was used to align all trajectories to their corresponding starting structures. The root-mean-square deviation (RMSD) values of all backbone atoms with respect to the initial conformation were calculated by VMD [42], and their average values were used to determine the overall backbone dynamics. The snapshot closest to the average structures was used as a representative of each simulation. The root-mean-square fluctuation (RMSF) of all protein residues with respect to their average position was calculated with VMD [42] and used to analyze protein residue flexibility. The conformational change of XfOhr in the simulation was followed by measuring the distance between the Arg19-Cα and C_p_-Cα/Glu51-Cα atoms throughout the simulation time using VMD [42]. For the residues Arg19, Glu51 and C_p_, the stability of the hydrogen bond interactions was measured by hydrogen bond (Hbond) occupancy throughout the entire trajectory using the default parameters of the VMD hydrogen bond tool (donor-acceptor distance and angle values of 3.0 Å and 20°, respectively). The stability of the salt-bridge interactions between these residues was measured considering the distance between all N–O/S pairs throughout the simulation using VMD [42]. These distances were analyzed by Tukey box-plots generated by R [43], and only residues having at least one N–O/S pair whose median distance value was lower than 4 Å [44, 45] were considered to be stable. PyMOL [26] and VMD [42] were used for visualization of both the trajectories and the representative structures. MD simulation movies were generated using UCSF Chimera [28].

## Results

### Crystal structure of XfOhr as a disulfide in its open form

Ohr proteins contain a distinct α/β fold, and there are currently only six structures deposited in the Protein Data Bank. Therefore, it is relevant to make new Ohr crystal structures available for comparative studies. Two of these structures are of XfOhr, and both are in the closed state [11]. A new structure of XfOhr in the open state is described here.

XfOhr was crystallized by the hanging drop vapor diffusion method, and the corresponding crystal belongs to space group C2 (Table 1). A complete data set was collected up to 2.15 Å resolution. The molecular replacement solution contained three monomers in the asymmetric unit. As expected from the previous characterizations [6,11], the overall structure of XfOhr in the disulfide state is an elliptically shaped homodimer. The superposition of the two XfOhr structures (PDB entries 4XX2 and 1ZB8/1ZB9) resulted in an RMSD = 1.21 Å. The XfOhr open-state structure was obtained in the oxidized state (disulfide bond) despite the presence of a reducing agent (dihydrolipoamide) in the solution. It is possible that the growth of the crystal started after the oxidation of dihydrolipoamide. Nevertheless, the same phenomenon occurred with DrOhr, but in this case, DTT instead of dihydrolipoamide was used as the reducing agent [15]. It is well documented that the efficacy of thiols as reductants decreases over time [46].

**Table 1:** Data collection and refinement statistics parameters for the XfOhr open-state.

Parameter	XfOhr open state
I. Data Collection	
• Space group	C2
• Unit-cell dimensions (Å)	*a* = 87.81; *b* = 83.69; *c* = 60.76
• Unit-cell angles (°)	α = γ = 90 and β = 93.67
• Resolution limits (Å)	43.81–2.15
• Total no. reflections	229678
• No. unique reflections	25723
• Completeness (%)	99.9 (99.9)
• Multiplicity	3.1 (3.0)
• R sym (%)	0.088 (0.349)
• < I/σ(I)>	13.9 (3.0)
II. Refinement statistics	
• Reflections	23868
• Working	22647
• Test	1221
Non-hydrogen atoms	3394
No. of water molecules	435
R_*factor*_	0.176
R_*free*_	0.223
RMDS values	
Bonds	0.001
Angles	1.539
• Average B-factor	
Main chain	20.24
Side chains and water molecules	22.44
• Ramachandran analysis (%)	
Favored regions	91.9
Additionally allowed regions	8.1
• PDB code	4XX2

**Fig 1:**
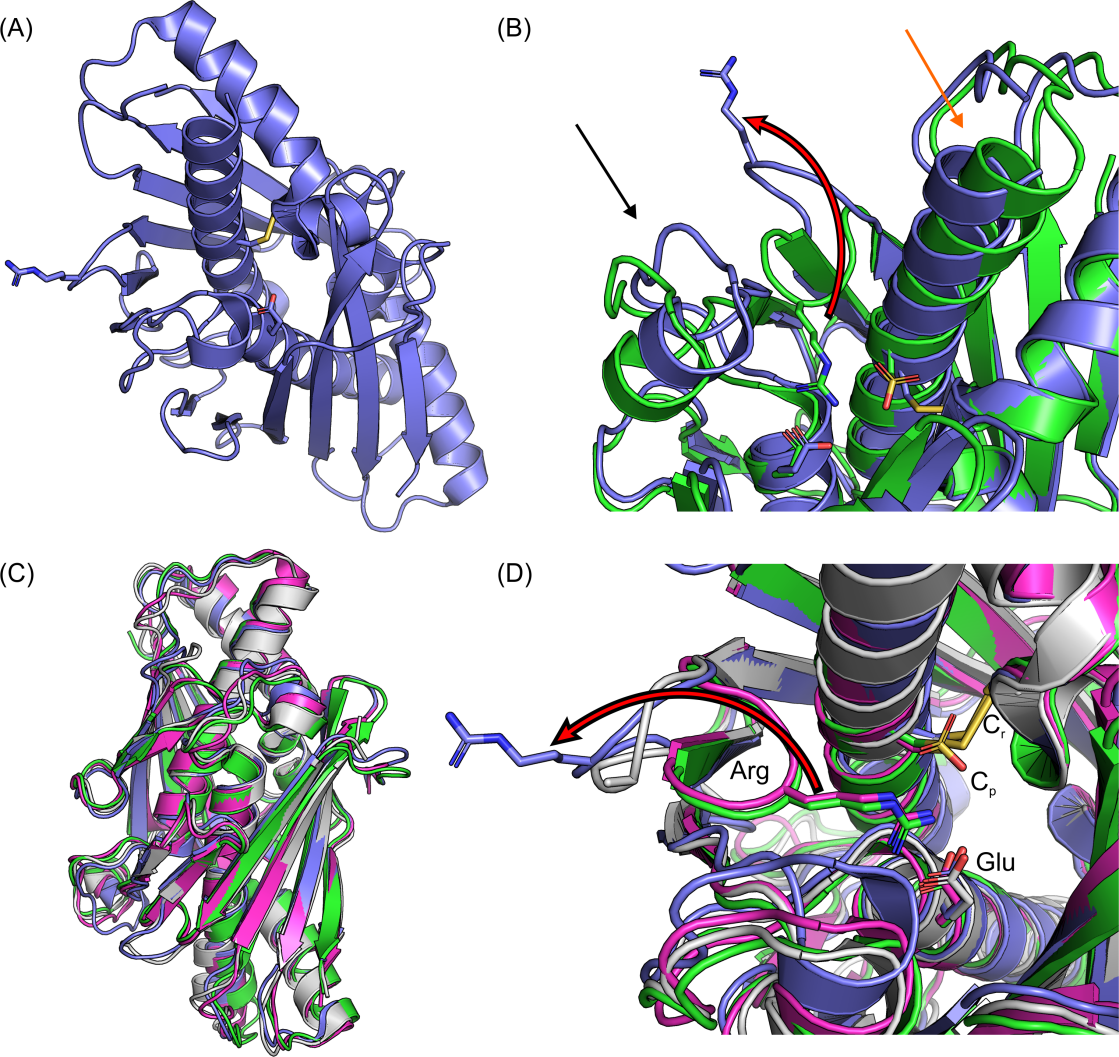
Comparison of different crystal structures of Ohr. (A) XfOhr open-state crystal structure in its oxidized form (Arg19 loop exposed to the solvent). (B) Superposition of the XfOhr open (green) and closed (blue marine) structures. The red arrow shows a shift between the open and closed conformations of the alpha helix containing C_p_ (moves approximately 2.1 Å), and the black arrow shows the superposition of the loop containing residues 33 to 48 (Gly-rich loop). (C) Superposed structures of XfOhr closed (blue marine); XfOhr open (green); PaOhr closed (pink) and DrOhr open (gray). (D) Active site of the XfOhr open (blue marine) and closed (green) states superimposed onto PaOhr closed (pink) and DrOhr open (gray) states. All backbone atoms are shown in cartoon representation, and the active site Arg (Arg19 in XfOhr), Glu (Glu51 in XfOhr), C_p_ (Cys61 in XfOhr) and C_r_ (Cys125 in XfOhr) residues are shown in stick representation. PDB entries: XfOhr closed (1ZB8); XfOhr open (4XX2); PaOhr closed (1N2F) and DrOhr open (1USP) states.

In the XfOhr open-state structure, the two fully conserved Cys residues are linked by a disulfide bond (Cys61-S-S-Cys125, S1 Fig), and the Arg19 loop is displaced far away from C_p_ (Cys61) in an open configuration (Fig 1A), in contrast to the reported XfOhr closed-state structure (Fig 1B) [11]. Other differences between the two XfOhr states are: (i) the α-helix that contains C_p_ is slightly bent in the open form and (ii) a Gly-rich loop containing residues 35 to 46 (Fig 1B), which is referred herein as the Gly-rich loop. In spite of these differences, the overall fold of XfOhr is quite similar to that of other Ohr structures (Fig 1C).

The main chains of the Arg19 loops of the XfOhr open state and of DrOhr overlapped well (Fig 1C). In the case of DrOhr, it was not possible to assign electronic densities to the side chains of the Arg19 loop [15], whereas the corresponding assignment for XfOhr was possible (S1 Fig), probably due to crystal contacts (S2 Fig). Possibly, the Arg19 side chain position observed in the XfOhr open structure may differ from the biological structure. Nevertheless, the XfOhr open state structure shares several structural features with DrOhr.

In the other structure, XfOhr is in the closed state and Arg19 makes polar interactions with Glu51 and with C_p_ (Fig 1D). The opening of the loop would probably then be facilitated by the loss of some of the polar interactions that occurred when C_p_ was oxidized to Cys-SOH, subsequently forming an intramolecular disulfide bond with the C_r_ (Fig 1A).

**Fig 2:**
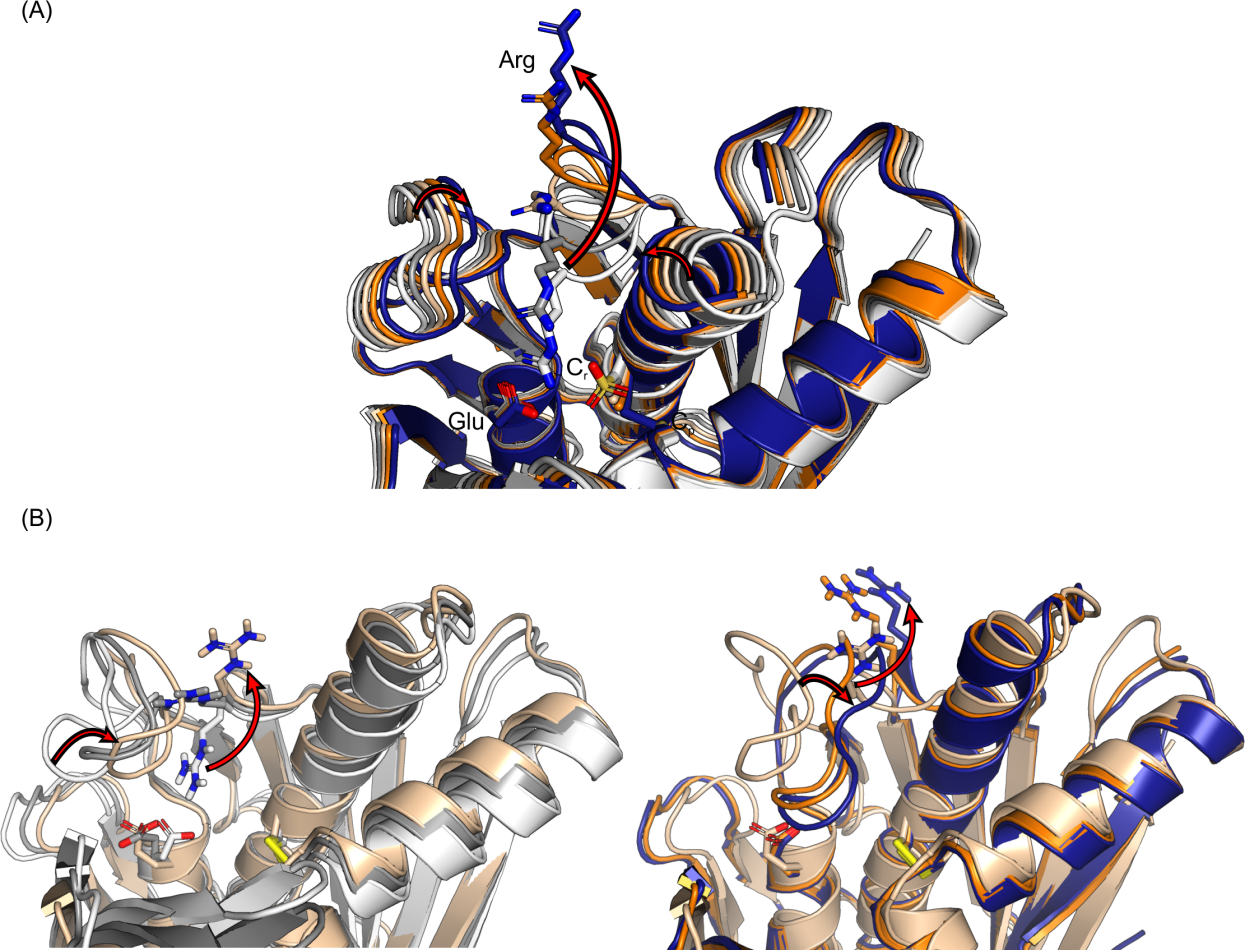
Morph conformations superimposed to the closed and open states of XfOhr. (A) Morph conformations generated using the two crystal structures of XfOhr in its closed (1ZB8) and open states (4XX2). The XfOhr crystal structure in its closed and open states is shown in white and blue, respectively. Gray, beige and orange structures correspond to some of the morph conformations generated by Chimera. (B) Morph conformations generated using the first, the average and the last snapshots of the XfOhr-SS trajectory. The XfOhr first and last snapshots are shown in white and blue, respectively. Gray, beige and orange structures correspond to some of the morph conformations generated by Chimera. This figure was divided in two, which represents two views in distinct orientations of the same movements. The red arrows indicate the major movements observed from the closed to the open state, namely, the Arg19 loop moves away from the active site, the Gly-loop moves into the active site and the shift of the alpha helix containing C_p_.

To gain insights into the conformational changes between the open and closed states, we made a morph conformations movie using 1ZB8 (open) and 4XX2 (closed) as the reference XfOhr crystal structures (see Fig 2A and S1 Video). This morph conformations movie suggested a concerted movement involving the Arg19 and Gly-rich loops. While the Arg19 loop moved far away from the XfOhr active site, the Gly-rich loop occupied this space, which is near the active site.

### Molecular dynamics of XfOhr: From closed to open states

To evaluate the role of polar interactions in the structural movements of the Arg19 loop, MD simulations were performed after “*in silico*” reconstruction of the disulfide bond between its C_p_ and C_r_ in the XfOhr closed state structure. With this disulfide bond, no polar interactions between C_p_ and Arg19 can occur, and therefore, some of the constraints on the dynamics of the Arg19 loop were relieved (S3 Fig). As a control, the XfOhr closed state in the reduced form (Cys61 as thiolate) was also subjected to MD simulation. Both of these simulations were performed for 150 ns. These two XfOhr simulations in the oxidized and reduced forms of the closed state structure are referred to herein as XfOhr-SS and XfOhr-S^-^, respectively. The XfOhr-S^-^ trajectory displayed an average RMSD = 2.14 ± 0.31 Å, indicating that its overall fold was stable [47] throughout the entire simulation (Table 2). In contrast, the average RMSD value for XfOhr-SS was 3.24 ± 0.50 Å, which was consistent with some conformational changes taking place. Notably, the RMSD values of the XfOhr-SS structure increased rapidly in the first 30 ns, reaching values up to 4 Å (S4 Fig).

**Table 2:** Average backbone RMSD (Å) and standard deviation values with respect to the corresponding starting structures calculated for each simulation.

Backbone atoms of	Simulation					
	XfOhr–S^-^	XfOhr-SS	XfOhr-SH	E51A	R19A	Open-S^-^
All protein	2.14 ± 0.31	3.24 ± 0.50	2.37 ± 0.43	3.07 ± 0.54	2.81 ± 0.44	3.12 ± 0.37

**Fig 3:**
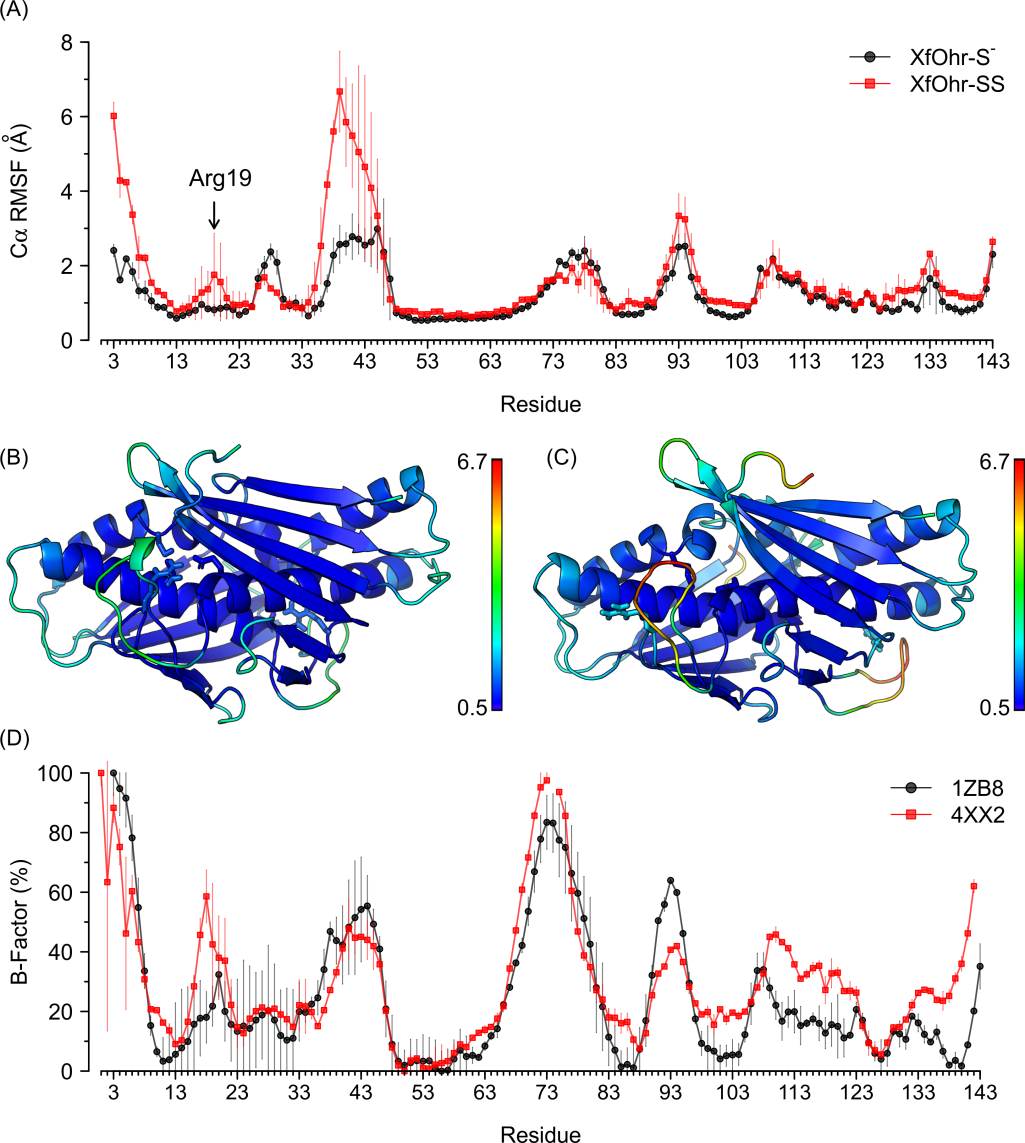
Localized fluctuations for XfOhr in the reduced and oxidized states. (A) Plot of Cα RMSD per-residue average values (Å) of both chains for XfOhr-S- (black) and XfOhr-SS (red) trajectories; standard deviations are shown as vertical lines. (B) XfOhr-S^-^ representative structure. (C) XfOhr-SS representative structure. In (B) and (C), the protein backbone atoms and the Arg19, Glu51, Cys61 and Cys125 residue side chains are shown in cartoon and in stick representations, respectively. All protein atoms are colored by their corresponding Cα RMSF values, ranging from 0.5 Å (blue) to 6.7 Å (red), as indicated by the right side bars. The snapshot closest to the average structure of each simulation was used as its representative structure. (D) Plot of normalized per-residue B-factors for the 1ZB8 (black) and 4XX2 (red) structures. Per-residue B-factors were calculated by averaging all Cα atom B-factors for both structures, separately. These total averages were normalized, where 0 and 100% correspond to the smallest and the largest averages, respectively. The vertical lines depict the corresponding standard deviations. The Cα atom B-factors for 1ZB8 and 4XX2 were determined using two monomers of each homodimer.

Next, we calculated the per-residue Cα RMSF to measure more localized fluctuations along the simulations. For the Arg19 residue, the values were approximately 1.0 and 1.8 Å during the XfOhr-S^-^ and XfOhr-SS trajectories, respectively (Fig 3A). The higher values observed for the XfOhr-SS trajectory than those of the XfOhr-S^-^ trajectory indicated that the Arg19 loop underwent conformational movements in the first case (Fig 3A–3C). Notably, the X-ray diffraction data (B-factors) also indicated that the Arg19 loop displayed a higher mobility in the open state than in the closed state (Fig 3D). Although the correlation of the intensities between the MD simulations and the X-ray data is not perfect, the overall profile of peaks and valleys displayed high correspondence (Fig 3A and 3D).

The Arg19 loop was not the region that presented the highest RMSF values. Instead, the residues from positions 33 to 48 (KLSVPQGLGGPGGSGT) in both simulations displayed the highest RMSF values, which is consistent with the fact that this loop (the Gly-rich loop) is mainly composed of short side chain residues (Fig 3A–3C). Residues 71 to 82 and 88 to 98 also displayed a higher mobility than the Arg19 loop (Fig 3A–3C). Moreover, the high flexibility of these three regions was also experimentally observed (*c*.*f*., B-factors of Fig 3D).

For further analysis of the dynamics of these loops along the XFOhr-SS trajectory, we made a morph conformations movie using the starting, the average and final conformation observed during this trajectory (Fig 2B and 2C and S2 Video). From this analysis, we again observed a concerned movement between Arg19 and the Gly-rich loops. According to the previous results (Fig 3), the Gly-rich loop movement was more pronounced than the Arg19 loop movement.

**Fig 4:**
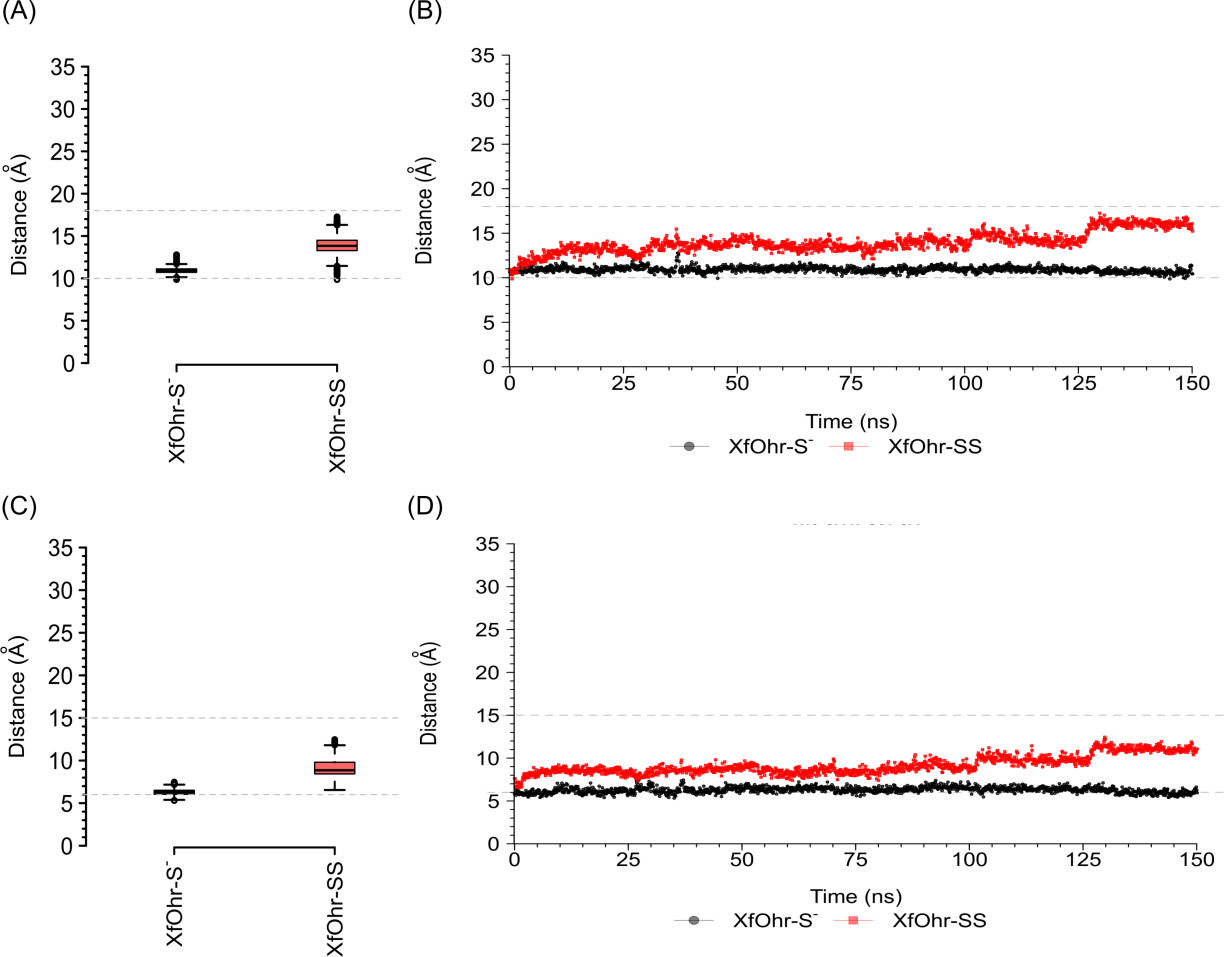
**Distance values between Arg19-Cα and Glu51-Cα atoms (A and B) and between Arg19-Cα and Cp-Cα atoms (C and D) for the XfOhr-S**^**-**^ **(black) and XfOhr-SS (red) trajectories (150 ns each).** Distance value distributions are shown as Tukey box-plots for Arg19—Glu51 (A) and Arg19—Cp (C) distances. In the Tukey box-plots (A and C), boxes indicate the interquartile distances, black lines show the median values, whiskers extend the box to 1.5 times the interquartile distance and circles represent outliers (values higher/lower than the whiskers). The box size shows the spread of the distance values, *i*.*e*., small boxes indicate less spread in the distance values. XfOhr-S^-^ (black) Arg19—Glu51 and Arg19—Cp median distance values are equal to 11 and 6 Å, respectively. XfOhr-SS (red) Arg19—Glu51 and Arg19—Cp median distance values are equal to 14 and 9 Å, respectively. The distance values are also shown as a function of simulation time for Arg19—Glu51 (B) and Arg19—Cp (D) distances. The average distance values between Arg19—Glu51 and Arg19—Cp Cα atoms obtained for the closed state (PDB entry = 1ZB8) and the open state (PDB entry = 4XX2) are shown as gray dashed lines at 10/6 and 18/15 Å, respectively.

Due to its importance for catalysis, the movement of the Arg19 loop was further analyzed by measuring the distances of the Arg19-Cα atom to the C_p_-Cα and Glu51-Cα atoms throughout the simulations (Fig 4). For comparison, the average distance of Arg19-Cα to Glu51-Cα was 10 Å in the closed-state crystal structure and 18 Å in the open-state crystal structure. Likewise, the average distance between Arg19-Cα and C_p_-Cα was 6 Å and 15 Å in the crystals structures in the closed and open states, respectively. The XfOhr-S^-^ trajectory appears to be more stable in a conformation more similar to the closed-state crystal structure of XfOhr, whereas the XfOhr-SS trajectory displayed more freedom with intermediate distances between the two crystal structures (Fig 4). These findings further suggested that the Arg19 loop was less constrained in the XfOhr-SS state, being able to move away from the active site. In contrast, the Arg19 loop kept its position close to C_p_ throughout the entire XfOhr-S^-^ simulation.

**Fig 5:**
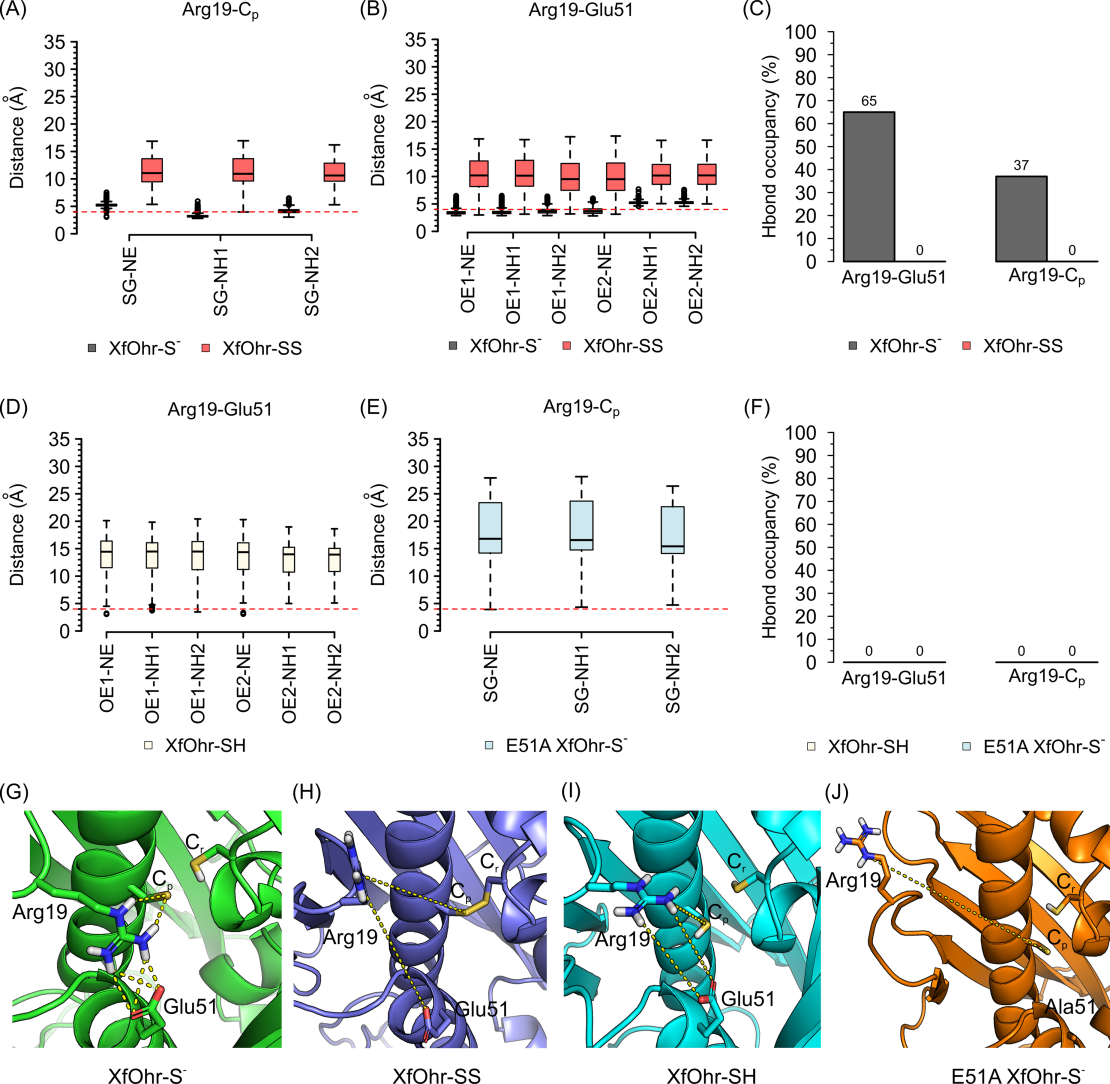
Salt-bridge and Hbond interactions of Arg19 with C_p_ and with Glu51 during XfOhr-S^-^ (black), XfOhr-SS (red), XfOhr-SH (beige) and E51A XfOhr-S^-^ (light blue) simulations. Distance value distributions are shown as a Tukey box-plots (A,B,D,E), in which boxes indicate the interquartile distances, black lines show the median values, whiskers extend the box to 1.5 times the interquartile distance and circles represent outliers (values higher/lower than the whiskers). The box size shows the spread of the distance values, *i*.*e*., small boxes indicate less spread in the distance values. The red dashed lines show the 4 Å cutoff value used as the criterion to define stable salt-bridge interactions [45]. The occupancy values for the Hbond interactions throughout the simulations are presented as bar plots (C,F). (A) Distances between the gamma sulfur atom (SG) of C_p_ and the nitrogen (NE, NH1, NH2) atoms of the Arg19 guanidinium group for XfOhr-S^-^ (black) and XfOhr-SS (red) simulations. (B) Distances between the oxygen atoms of Glu51 (OE1, OE2) and nitrogen (NE, NH1, NH2) atoms of the Arg19 guanidinium group for XfOhr-S^-^ (black) and XfOhr-SS (red) simulations (150 ns each). (C) Occupancy values of Hbond interactions between Arg19—Glu51 and Arg19—Cp during the XfOhr-S^-^ (black) and XfOhr-SS (red) simulations (150 ns each). (D) Arg19 –Glu51 salt-bridge interaction distance values measured during the XfOhr-SH simulation (50 ns), considering all N–O pairs described in B. (E) Arg19 –C_p_ salt-bridge interaction distance values measured during the E51A XfOhr-S^-^ simulation (50 ns), considering all N–S pairs described in A. (E) Occupancy values of Hbond interactions between Arg19—Glu51/Ala51 and Arg19—C_p_ during the XfOhr-SH (beige) and E51A XfOhr-S^-^ (light blue) simulations (50 ns each). Distance values (yellow dashed lines) measured for the representative structures: (F) XfOhr-S^-^ (green, values = 2.2–3.7 Å), (G) XfOhr-SS (blue marine, values = 11.9 and 13.1 Å), (H) XfOhr-SH (cyan = 8.0–9.0 Å) and (I) E51A XfOhr-S^-^ (orange, value = 19.0 Å). Each representative structure corresponds to the MD simulation snapshot closest to the average structure calculated for its trajectory (50 or 150 ns). The protein backbone atoms are show in cartoon representation, and the 33–48 loop was removed for clarity. Arg19, Glu51, C_p_ and C_r_ side chain atoms are shown in stick representation, including their polar hydrogen atoms.

The polar interactions among C_p_—Arg19—Glu51 residues were further investigated by analyzing the distances involving atoms of the side chains (Fig 5A and 5B). As expected, the Arg19—Glu51 and Arg19—Cys61 salt-bridge interactions were stable in the XfOhr-S^-^ trajectory (median values <4 Å for nearly all N–O/S pairs, *c*.*f*., Material and Methods). Likewise, the Arg19—Glu51 and Arg19—Cys61 hydrogen bond (Hbond) interactions were observed throughout the XfOhr-S^-^ trajectory, with occupancy values equal to 65 and 37%, respectively (Fig 5C). In contrast, these interactions were unstable or even absent during the XfOhr-SS trajectory (median values > 4 Å for all N–O/S pairs and hydrogen bond occupancy values = 0%, Fig 5C). Indeed, the corresponding median values of the Arg19—Glu51 and Arg19—Cys61 distances for XfOhr-SS trajectories were high, reaching values of approximately 10 Å (Fig 5A and 5B).

**Fig 6:**
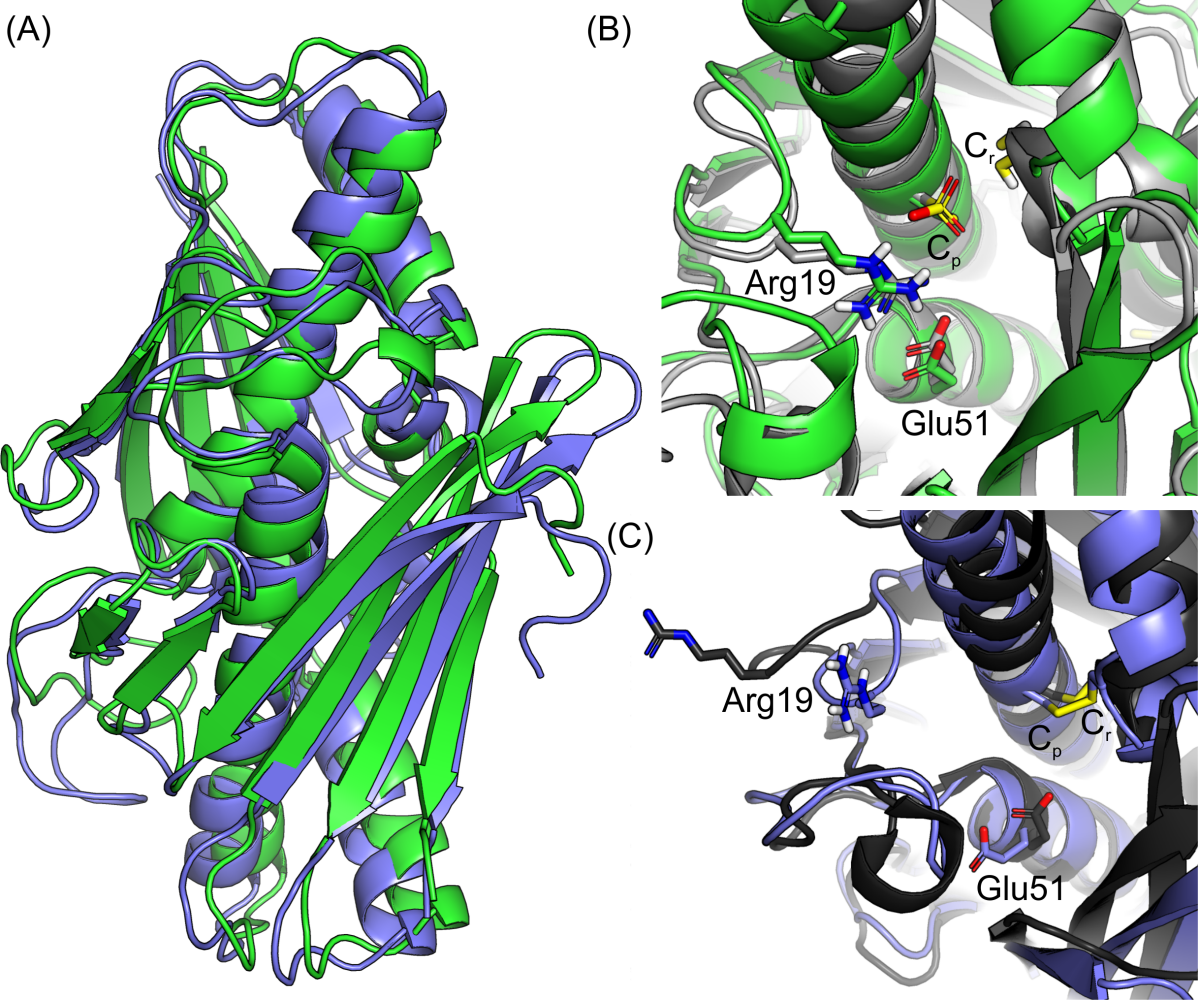
XfOhr-S^-^ and XfOhr-SS representative structures from MD simulations. (A) Comparison between the overall representative structures of XfOhr-S^-^ (green) and XfOhr-SS (blue marine). (B) Active site of the XfOhr-S^-^ representative structure (green) superposed to the XfOhr crystal structure (light gray) in its closed state (PDB entry 1ZB8). (C) Active site of the XfOhr-SS representative structure (blue marine) superimposed to the XfOhr crystal structure (dark gray) in its oxidized form and open state (PDB entry 4XX2, described in this paper). Each representative structure corresponds to the MD simulation snapshot closest to the average structure calculated throughout the entire trajectory (150 ns). The protein backbone atoms are shown in cartoon representation, and the side chains atoms of Arg19, Glu51, C_p_ and C_r_ are shown in the stick representation.

**Fig 7:**
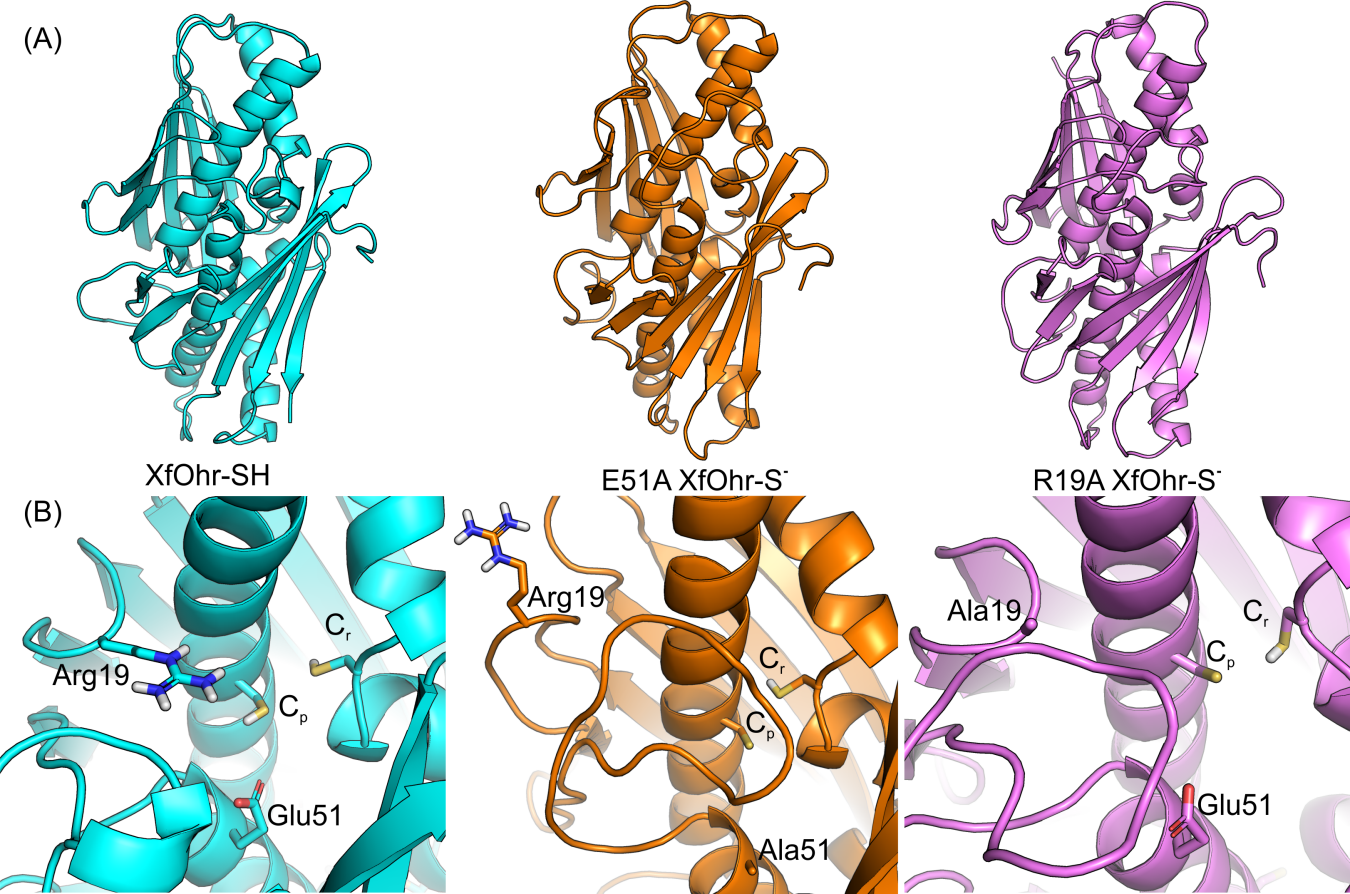
Representative structures of XfOhr-SH, XfOhr mutants from MD simulations. (A) Overall representative structure of the XfOhr-SH (cyan), E51A-XfOhr (orange) and R19A-XfOhr (purple) simulations. (B) Active sites of representative structure of the XfOhr-SH (cyan), E51A-XfOhr (orange) and R19A-XfOhr (purple) simulations. Each representative structure corresponds to the MD simulation snapshot closest to the average structure calculated for its trajectory (50 ns). The protein backbone atoms are show in cartoon representation and Arg19/Ala19, Glu51/A1a51, C_p_ and C_r_ side chain atoms are shown in stick representation.

The snapshot closest to the average structure throughout each MD simulation was used to represent the entire XfOhr-SS and XfOhr-S^-^ trajectories (Fig 6). No significant difference in the overall structure was observed between the two average structures (Fig 6A, with RMSD = 1.93 Å). Despite these similarities, their Arg19 loops adopted two different conformations. In the XfOhr-S^-^ representative structure, the Arg19 loop adopted a closed orientation in both chains, which were very similar to the closed crystal structure (Fig 6B and S3 Video). Furthermore, Arg19 kept stable hydrogen bond and salt-bridge interactions with Glu51 and C_p_ (both identified as stable during the entire XfOhr-S^-^ simulation). In contrast, the Arg19 loop of the XfOhr-SS representative structure underwent a conformational change, moving away from the active site, which was similar to the conformation observed in the open crystal structure (Fig 6C and S4 Video). Both chains adopted this open state; however, their Arg19 loop orientations were somewhat different (S5 Fig). Another difference observed between the XfOhr-SS and XfOhr-S^-^ representative structures is the Glu51 side chain orientation. In the XfOhr-S^-^ representative structure, the Glu51 side chain is oriented toward the guanidinium group of Arg19, establishing polar contacts (Fig 6B). On the other hand, in the XfOhr-SS representative structure, the Glu51 side chain is oriented toward the Gly-rich loop (Fig 7C). From a visual inspection of the Glu51 side chain movement along the XfOhr-SS trajectory, we observed that Glu51 initially formed polar contacts with Arg19. However, as Arg19 moved away from the XfOhr active site, the Arg19—Glu51 interactions were lost. As a result, the Glu51 side chain was less constrained, being able to establish hydrogen bond interactions with other residues, in particular with those of the Gly-rich loop. Probably, these changes observed in the Glu51 conformations are part of the concerted movement between the Arg19 and Gly-rich loop described previously (S2 and S4 Videos).

Next, we investigated whether the polar interactions between the Arg19 and C_p_ residues could restrict the movement of the corresponding loop. Therefore, another MD simulation (50 ns) was performed, again with XfOhr in the closed state, but now having both thiols artificially protonated. This simulation is referred to herein as XfOhr-SH. Since the Arg19 loop movement started immediately after the initial 30 ns in the XfOhr-SS trajectory (S4 Fig), we assumed that 50 ns of simulation would be enough to observe similar movement in the XfOhr-SH trajectory. Indeed, the Arg19 loop of both chains moved away from the active site, leaving the Arg19 side chain highly exposed to the solvent (Figs 5D, 5F, 5I and 7 and S5 Video). Furthermore, the Arg19 –Glu51 salt-bridges and hydrogen bond interactions were unstable during the XfOhr-SH trajectory (Fig 5D and 5F, respectively). Moreover, the distances between Arg19-Cα and Glu51-Cα were comparable with those observed for the XfOhr-SS simulation, with values up to 19 Å (S6 Fig). These findings further support the notion that the salt-bridge interaction between Arg19 and the negatively charged C_p_ (Cys61-S^-^) plays a relevant role in stabilizing the Arg19 loop near the active site. Thus, our simulations indicated that the Arg19 loop movement is constrained by polar interactions between C_p_-S^-^ and Arg19. Considering the overall XfOhr-SH simulation, the average RMSD values were in between those observed for the XfOhr-S^-^ and XfOhr -SS trajectories (Table 2).

Subsequently, we addressed whether Glu51 could also contribute to the stabilization of the XfOhr structure in the closed state. Thus, the E51A XfOhr-S^-^ mutant was artificially built and subjected to MD simulations (50 ns). Again, the Arg19 loop moved away from the active site (Figs 5E, 5F, 5J and 7 and S6 Video). In this case, the movement occurred in an even shorter interval than those observed for the XfOhr-SS and XfOhr-SH trajectories. The highest Arg19-Cα to Ala51-Cα distance observed during the E51A XfOhr-S^-^ trajectory was 27 Å (S7 Fig). Furthermore, the Arg19 –C_p_ salt-bridge and hydrogen bond interactions were also unstable during this trajectory (Fig 5E and 5F). Therefore, Glu51 is also crucial for stabilizing the Arg19 loop close to the active site, in this case independently of the C_p_ oxidative state.

Finally, we artificially built a R19A substitution in the XfOhr-S^-^ structure in the closed state. According to our hypothesis, the mutated “Ala19” loop would move away from the active site. The median distance values for Ala19-Cα - Glu51-Cα and Ala19- Cα - C_p_- Cα distance values were equal to 16 and 12 Å, respectively (S8 Fig and S7 Video). Interestingly, these values were slightly shorter than those obtained for the E51A XfOhr-S^-^ mutant. The more restricted behavior of the R19A mutant could be related to the aliphatic side chain that confers hydrophobic properties to the Ala residue. As a consequence, polar interactions between the Ala19 side chain and solvent water molecules are not favorable, destabilizing the open-state conformation (S9 Fig).

### Molecular dynamics of XfOhr: From the open to closed states

Since our MD simulations indicated that upon C_p_ oxidation, XfOhr could move from the closed to the open state, we decided to verify whether the opposite process could occur, *i*.*e*., if the reduction of the Cys61-Cys125 bond would lead to the reverse movement (from the open to the closed state). In this case, the XfOhr open state was used as a starting point for MD simulation (150 ns), being named the Open XfOhr-S^-^ trajectory. Thus, the disulfide bond was artificially reduced, considering C_p_ (Cys61) and C_r_ (Cys125) as a thiolate (RS^-^) and a thiol (RSH), respectively.

Unexpectedly, the Arg19 loop did not undergo major movements but remained in its open state throughout the entire trajectory (S8 Video). Indeed, the observed distances of the Open XfOhr-S^-^ structure were similar to those measured in the XfOhr open-state crystal structure (S10 Fig). One hypothesis for this is that the crystal packing contacts in the crystal structure of the XfOhr open state (4XX2) artificially kept the Arg19 loop orientation more exposed to the solvent than in its native condition. Therefore, the starting structure used for the Open XfOhr-S^-^ trajectory would have the Arg19 loop orientation more distant from the active site than the biological one, preventing the closure of the Arg19 loop. Alternatively, entropic factors related to the dehydration of the Arg19 loop may have also prevented XfOhr from assuming the closed state. Indeed, the Ohr active site is surrounded by hydrophobic residues [11]. Furthermore, the Gly-rich loop (comprising residues 33–48) might have impaired the closure of the Arg19 loop. Our MD simulations are consistent with this possibility, as the Gly-rich loop appears to prevent the movement of the Arg19 loop back to the active site by steric hindrance effects (S8 Video), at least during the simulation time employed (150 ns). Further studies are required to understand the possible roles of the Gly-rich loop in catalysis.

### Biochemical analysis of XfOhr mutants (R19A and E51A)

**Fig 8:**
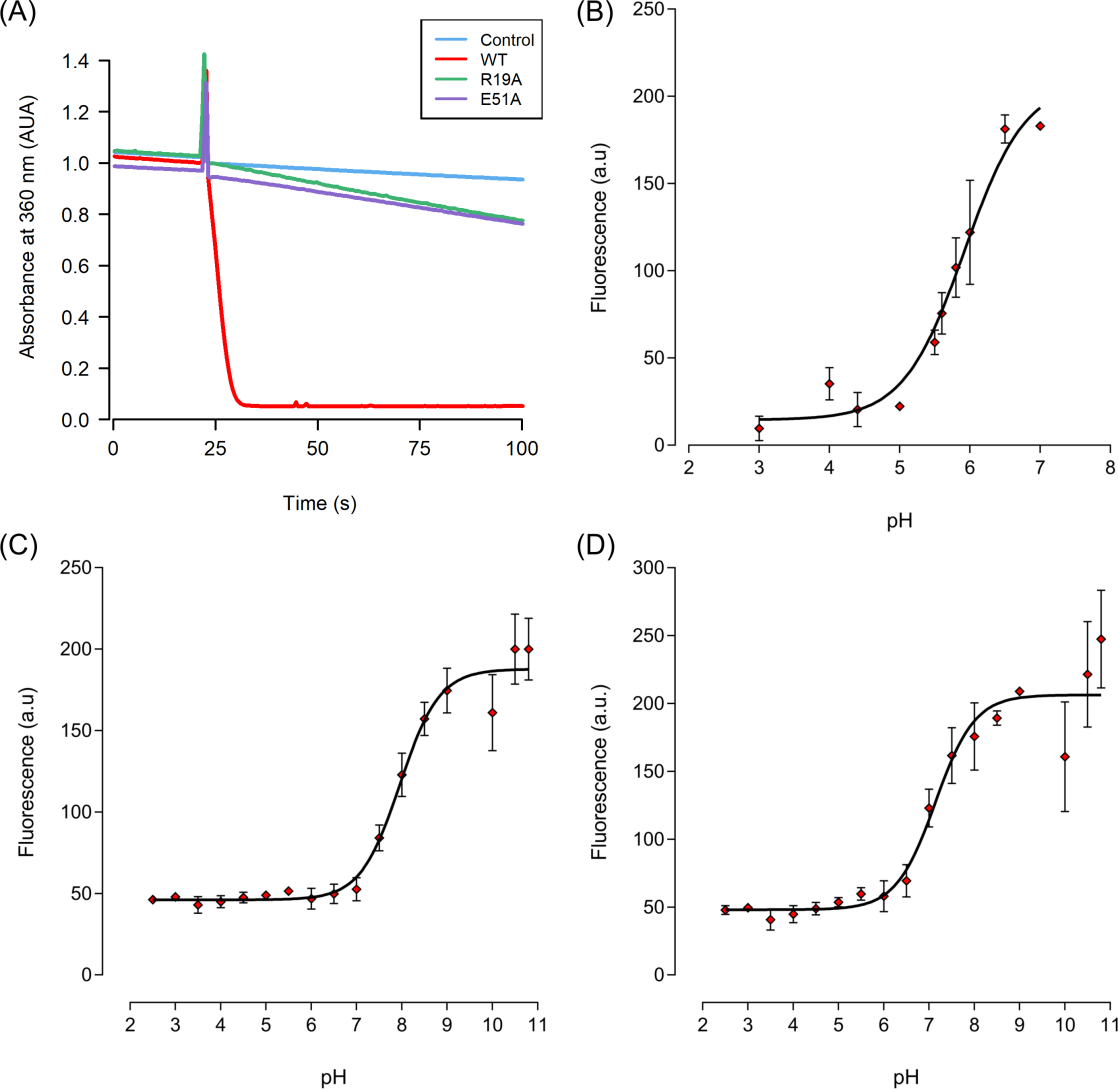
Comparative analyses of wild-type XfOhr and two mutants (R19A and E51A). (A) Lipoamide-lipoamide dehydrogenase peroxidase-coupled assay of wild-type XfOhr, R19A and E51A. The peroxidase activities were monitored by the oxidation of NADH at 340 nm in the presence of XfOhr (0.05 μM), lipoamide dehydrogenase from *X*. *fastidiosa* (XfLpD, 0.5 μM), and lipoamide (50 μM) in sodium phosphate buffer (20 mM, pH 7.4) and DTPA (0.1 mM). Cys61 (C_p_) pKa determination of wild-type XfOhr and two mutants (R19A and E51A) by the monobromobimane method; plots of fluorescence as a function of pH for wild-type XfOhr (B), R19A XfOhr (C) and E51A XfOhr (D). The red points show the mean values of at least two independent experiments. The error bars indicate the SEM. All pKa values were determined using the Henderson-Hasselbach equation of GraphPad^®^Prism4.

It is well accepted that the presence of Arg and Glu in the active site of Ohr in close proximity to C_p_ is an important factors for the high reactivity of this peroxidase toward hydroperoxides [1,6,11]. Furthermore, our MD simulation data presented here indicate that Glu51 and C_p_ are required to stabilize the Arg19 loop in the closed state. Therefore, two mutations (R19A and E51A) were generated by site-directed mutagenesis into the XfOhr recombinant protein to experimentally validate these *in silico* findings. As expected, compared with the wild-type protein, XfOhr R19A and XfOhr E51A presented only residual dihydrolipoamide-dependent peroxidase activity (Fig 8A). Moreover, these mutations resulted in significant changes in the pK_a_ of the catalytic Cys thiolate 8.09 (± 0.11) for R19A and 7.20 (± 0.11) for E51A) compared that observed in the wild-type protein (5.92 ± 0.11; Fig 8B to 8D). Previously, we also observed that wild-type XfOhr displays an acidic pK_a_ [48]. Here, we show for the first time that mutation of the catalytic Glu impairs the enzymatic activity of Ohr. Previously, the relevance of the catalytic Arg was analyzed by mutation in PaOhr [6]. Therefore, these results are consistent with the proposed roles of Arg19 and Glu51 in catalysis, as well as with our MD simulations. Indeed, the thiolate pK_a_ values for the R19A and E51A mutants are more similar to that of free cysteine [49] than the pK_a_ values corresponding to the wild-type protein. Circular dichroism spectra of the wild-type and mutant proteins in the reduced and oxidized states were very similar, excluding the hypothesis that the R19A and E51A mutations might provoke major problems to the overall structure of XfOhr (S11 Fig).

## Discussion

**Fig 9:**
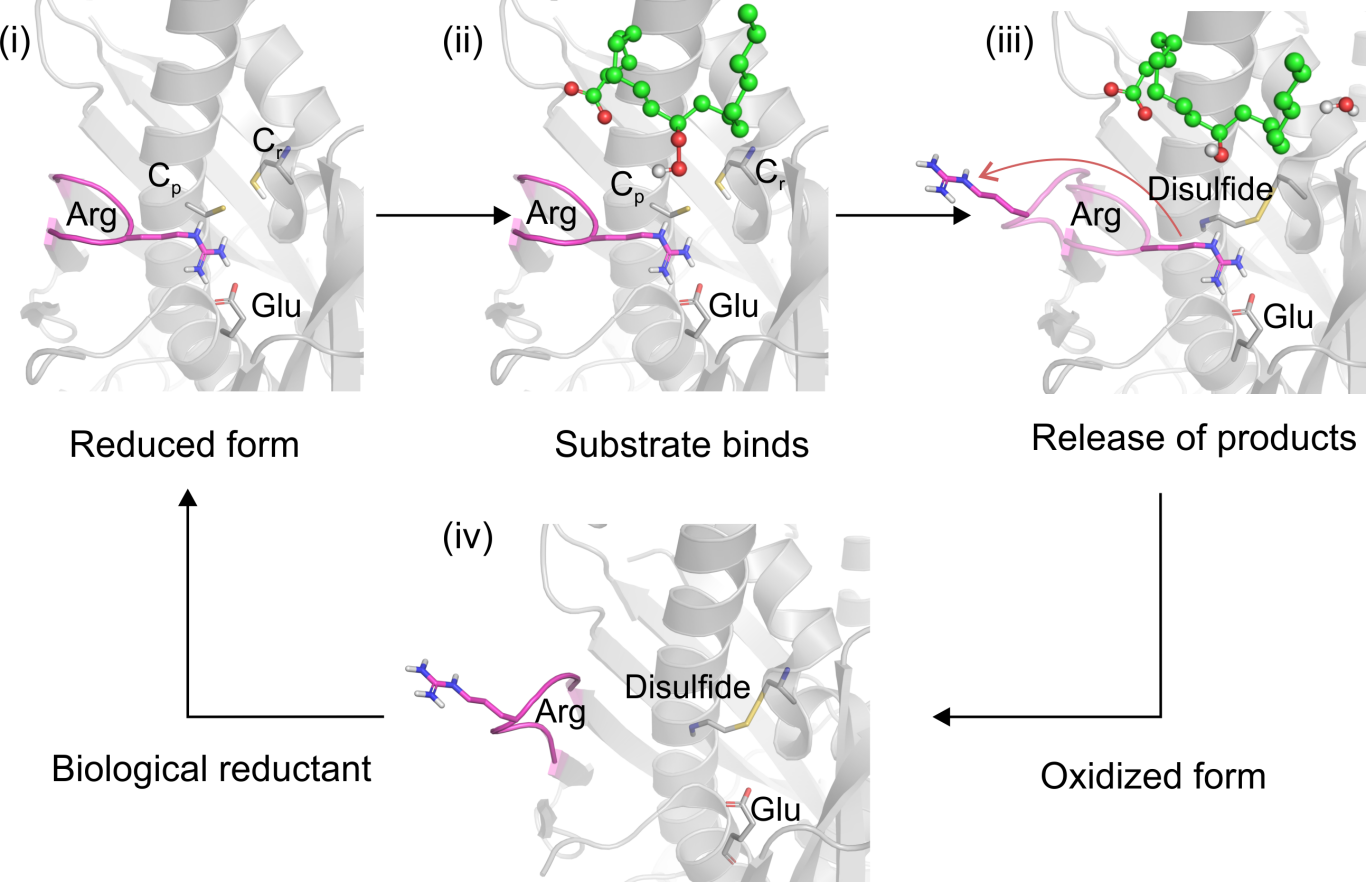
Proposed model for fatty acid hydroperoxide reduction by Ohr. (i) In the reduced form of Ohr (C_p_-S^-^, Cys61 of XfOhr), the thiolate anion (Sɤ of C_p_) makes an Hbond with the guanidinium group of the conserved Arg (Arg19 of XfOhr), which also makes a salt-bridge with the conserved Glu (Glu51 of XfOhr). (ii) The lipid hydroperoxide (LHP) is placed over the hydrophobic moiety of the Arg side chain, being also stabilized by other hydrophobic interactions. (iii) After peroxide reduction, C_p_ is oxidized to sulfenic acid (SOH), which is then attacked by the sulfhydryl group of the resolving Cys (Cys125 of XfOhr), forming an intra-molecular disulfide. Our working hypothesis is that this condensation reaction releases constraints for Arg19 loop movements. (iv) The last step involves the reduction of the disulfide by a lipoylated protein and a rearrangement of the loop to the close state (taken from [11]). Steps (ii) and (iii) are hypothetical, as substrate and product (respectively) were inserted based on the co-crystallization of PEG with XfOhr [11].

A working hypothesis for the catalytic mechanism of Ohr enzymes is presented in Fig 9. Most likely, the catalytic cycle of Ohr enzymes is more complex, and additional steps occur between the closed (Fig 9i) and open (Fig 9iv) states.

There are six crystal structures of Ohr enzymes deposited in the RSCB Protein Data Bank, and all of them are either in the open or in the closed state that corresponds either to snapshot (i) or (iv) in Fig 9, respectively. The closed state appears to be an optimal conformation for hydroperoxide reduction, as the catalytic Arg (Arg19 in XfOhr) is near to C_p_, which probably results in increased C_p_ nucleophilicity and ROOH electrophilicity. Indeed, in peroxiredoxins (another type of Cys-based peroxidase), a catalytic Arg plays a similar role [50]. In the open state, the entrance of the active site is wider [10], which might better accommodate lipoylated proteins that are the reducing agent of XfOhr [15]. Prior to this work, no information on intermediate states was available.

Initially, the opening of the Arg19 loop was investigated by MD simulations. The overall fold was stable throughout the XfOhr-SS simulation, but the Arg19 loop underwent an opening movement, among other conformational changes. In contrast, the Arg19 loop was stabilized in the closed form when C_p_ was reduced and unprotonated (Figs 4–6 and S3 Video), which is consistent with a pK_a_ for the thiolate group of C_p_ equivalent to 5.92 (Fig 9B). In contrast, when the thiol group of C_p_ was oxidized or protonated (Figs 4–8 and S6 Fig), Arg19 displayed greater freedom, moving away from the active site (Figs 6 and 7, S4 and S5 Videos). Therefore, according to our hypothesis, the stability of the Arg19 loop depends on the oxidative state of C_p_.

However, other factors also contribute to the stability of the Arg19 loop in the closed state, and a major one is the polar interaction of Arg19 with Glu51. Indeed, the mutation of Glu51 to Ala resulted in increased mobility of the Arg19 loop (Figs 5 and 7; S7 Fig and S6 Video), even when C_p_ was in the thiolate form. Therefore, the disruption of either the Arg19—C_p_ or the Arg19—Glu51 polar interaction facilitated the opening of the Arg19 loop.

We also performed MD simulations starting from the structure with the Arg19 loop in the open form in an attempt to investigate the closing of this loop. Contrary to our expectations, the Arg19 loop did not return to the closed state in any of the conditions and intervals analyzed (S10 Fig and S8 Video). Possibly, the removal of water molecules solvated to the Arg19 loop is required prior to the approximation of this region toward C_p_. One hypothesis is that the reducing agent (lipoylated proteins) might assist the closing process of the Arg19 loop. Another possibility is that this movement would require much longer simulation time (> 150 ns) or would be better observed using a different *in silico* technique, such as Normal Mode analysis [51]. Investigations in these directions are underway.

This is the first report that describes a biochemical feature associated with the Gly-rich loop (comprising residues from position 35 to 48) that was part of the XfOhr, which exhibited the greatest flexibility (Fig 3A). Hydrophobicity is another feature of the Gly-rich loop that is highly conserved among Ohr family members [7,12,14]. Remarkably, some of these residues interact with polyethylene glycol by hydrophobic interactions [11]. Indeed, docking studies [1] have indicated that the hydrophobic interactions are major factors for lipid hydroperoxide binding within the XfOhr active site. Interestingly, the position of the catalytic Arg considerably differs between Ohr and OsmC proteins that comprise two of the major sub-families in the Ohr/OsmC superfamily [14]. The catalytic Arg is in the Gly-rich loop in OsmC proteins. Therefore, our studies open new perspectives in the understanding of enzyme-substrate interactions in proteins belonging to the Ohr/OsmC superfamily, which may foster investigations aiming to identify inhibitors of these enzymes.

Furthermore, this study contributes information to help distinguish Ohr from other Cys-based peroxidases, such as Prx. Indeed, we have previously reported that Ohr and Prx display distinct biochemical and structural properties [1]. For instance, Ohr and Prx are not homologous proteins, as they do not share amino acid sequence or structural similarities [7,12,52]. For most Prx enzymes, the reductant is Trx, whereas for Ohr, the reductants are probably lipoylated proteins [10]. In this report, we present other features that distinguish Ohr from Prx enzymes. For instance, it is well known that Prx enzymes switch back and forth between the so-called fully folded and locally unfolded states when catalytic Cys residues undergo large movements to allow disulfide formation, as these two residues are far apart in the reduced state [53]. In contrast, the catalytic Arg remains relatively static throughout the catalytic cycle of Prx. In the case of XfOhr, the two catalytic Cys residues remain relatively static throughout the catalytic cycle, whereas the catalytic Arg19 undergoes movement between the closed and open states. Therefore, distinct mechanisms were selected throughout evolution that allowed for the development of two different systems, operating with extraordinary efficiency in hydroperoxide reduction and attaining rates in the 10^7^−10^8^ M^-1^ s^-1^ range [1,53,54].

Finally, it is important to emphasize that the high correspondence between the crystallographic and simulation data and the biochemical characterizations indicate the robustness of our analysis. Understanding catalytic cycle dynamics might be relevant for the development of Ohr inhibitors. Since Ohr enzymes are present in pathogenic bacteria and fungi [7] but are absent in their hosts, such as plants and animals, these enzymes might be promising targets for drug design.

## Accession codes

The crystal structure in this paper has been deposited in the Protein Data Bank as 4XX2.

## Supporting information

S1 Fig Active site residues and Arg19 loop density maps of XfOhr open-state crystal structures. (TIFF)

S2 Fig Crystalline contacts of XfOhr Arg19. (TIFF)

S3 Fig Active site surface colored by partial atom charges, as used in the MD simulations, ranging from +0.450 (blue) to -0.720 (red). (TIFF)

S4 Fig RMSD values of backbone atoms for XfOhr-S- (black) and XfOhr-SS (red) trajectories, with respect to their corresponding starting structures as a function of simulation time (ns). (TIFF)

S5 Fig Chain A (gray) superimposed to chain B (yellow) of the XfOhr-SS representative structure, showing a RMSD value of 1.24 Å.
(TIFF)

S6 Fig Distance values between Arg19-Cα and Glu51-Cα atoms and between Arg19-Cα and C_p_-Cα atoms for the XfOhr-SH trajectory (50 ns). (TIFF)

S7 Fig Distance values between Arg19-Cα and Ala51-Cα atoms and between Arg19-Cα and C_p_-Cα atoms for the E51A XfOhr-S- mutant trajectory (50 ns). (TIFF)

S8 Fig Distance values between Ala19-Cα and Glu51-Cα atoms and between Ala19-Cα and Cp-Cα atoms for the R19A XfOhr-S- mutant trajectory (50 ns). (TIFF)

S9 Fig Number of hydrogen bonds formed with Arg19/Ala19 during XfOhr-S- (dark gray), XfOhr-SS (red), XfOhr-SH (light blue), E51A XfOhr-S- (beige) and R19A XfOhr-S- (orange) trajectories (150 or 50 ns). (TIFF)

S10 Fig Distance values between Arg19, Glu51 and C_p_ for the Open XfOhr-S- trajectory (150 ns). (TIFF)

S11 Fig Circular dichroism spectra of XfOhr wild type, R19A and E51A. S1 to S8 Videos.
(TIFF)

S1 Video Morph conformations (orange) movie generated using the two crystal structures of XfOhr in its closed (1ZB8, white) and open (4XX2, blue) states. (MP4)

S2 Video Morph conformations (magenta) movie generated using the first (white), the average and the last (blue) snapshots of the XfOhr-SS trajectory. (MP4)

S3 Video XfOhr-S-_trajectory (150ns), showing the Arg loop and the Gly-rich loop in magenta and blue, respectively. (MP4)

S4 Video XfOhr-SS_trajectory (150ns), showing the Arg loop and the Gly-rich loop in magenta and blue, respectively. (MP4)

S5 Video XfOhr-SH_trajectory (50ns), showing the Arg loop and the Gly-rich loop in magenta and blue, respectively. (MP4)

S6 Video E51A XfOhr-S-_trajectory (50ns), showing the Arg loop and the Gly-rich loop in magenta and blue, respectively. (MP4)

S7 Video R19A XfOhr-S-_trajectory (50ns), showing the Arg loop and the Gly-rich loop in magenta and blue, respectively. (MP4)

S8 Video Open XfOhr-S-_trajectory (150ns), showing the Arg loop and the Gly-rich loop in magenta and blue, respectively. (MP4)
