# Supplementary figures and images for: Structural insights on the efficient catalysis of hydroperoxide reduction by Ohr: Crystallographic and molecular dynamics approaches
Source: PLoS One. 2018 May 21;13(5):e0196918. doi: 10.1371/journal.pone.0196918 (PMC5962072; doi:10.1371/journal.pone.0196918)

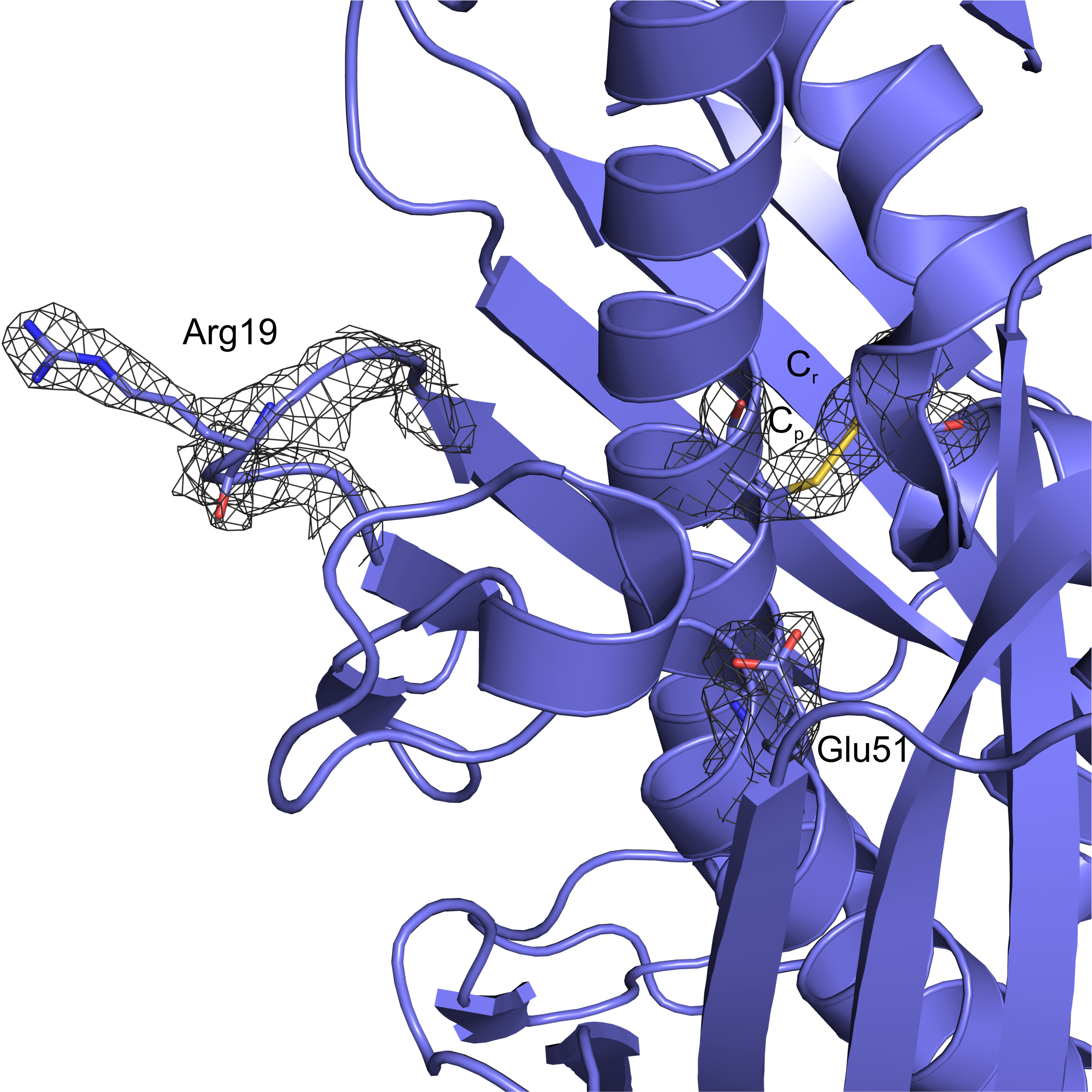

Supplement: S1 Fig — (TIFF) [file pone.0196918.s001.tiff]

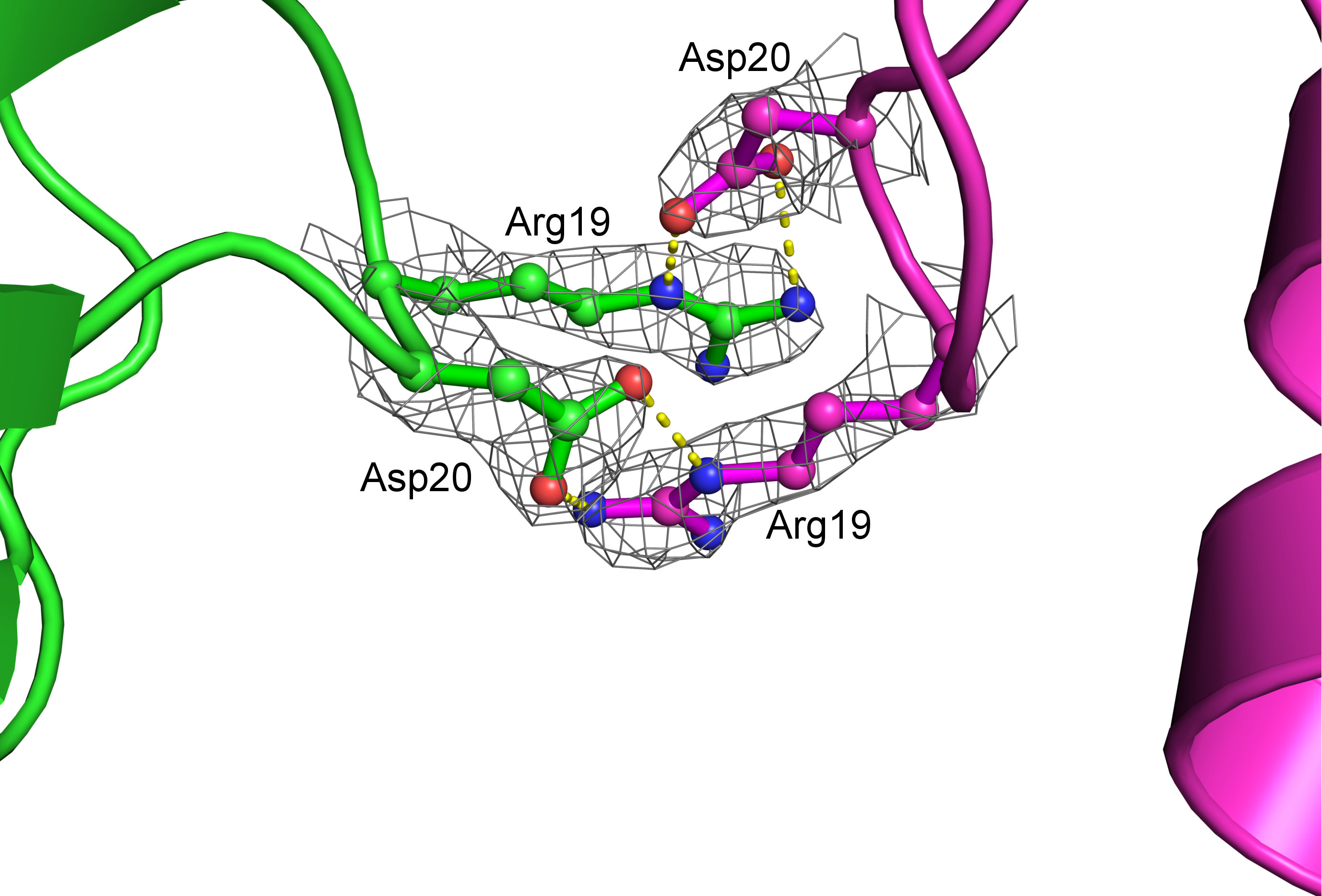

Supplement: S2 Fig — (TIFF) [file pone.0196918.s002.tiff]

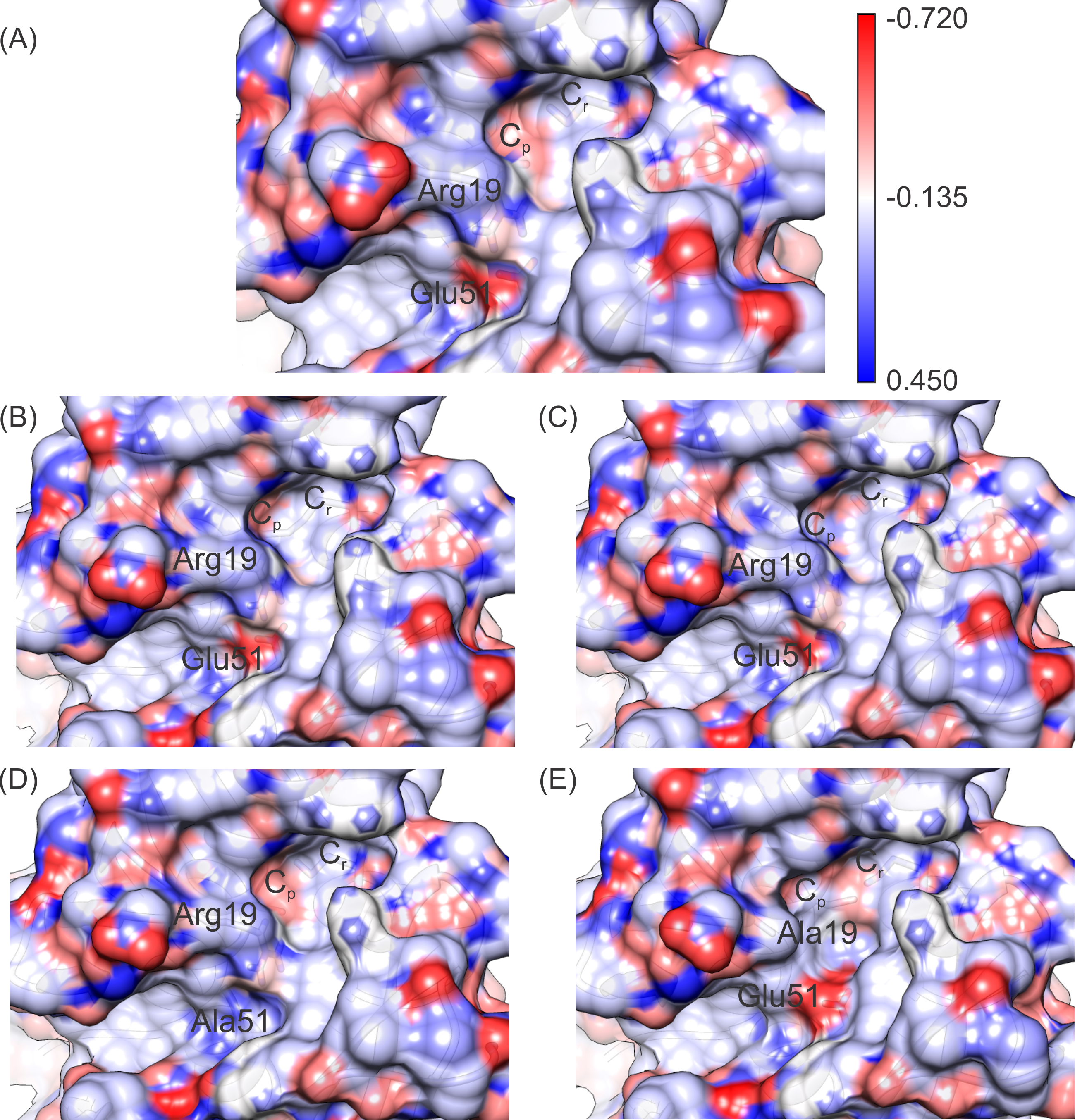

Supplement: S3 Fig — (TIFF) [file pone.0196918.s003.tiff]

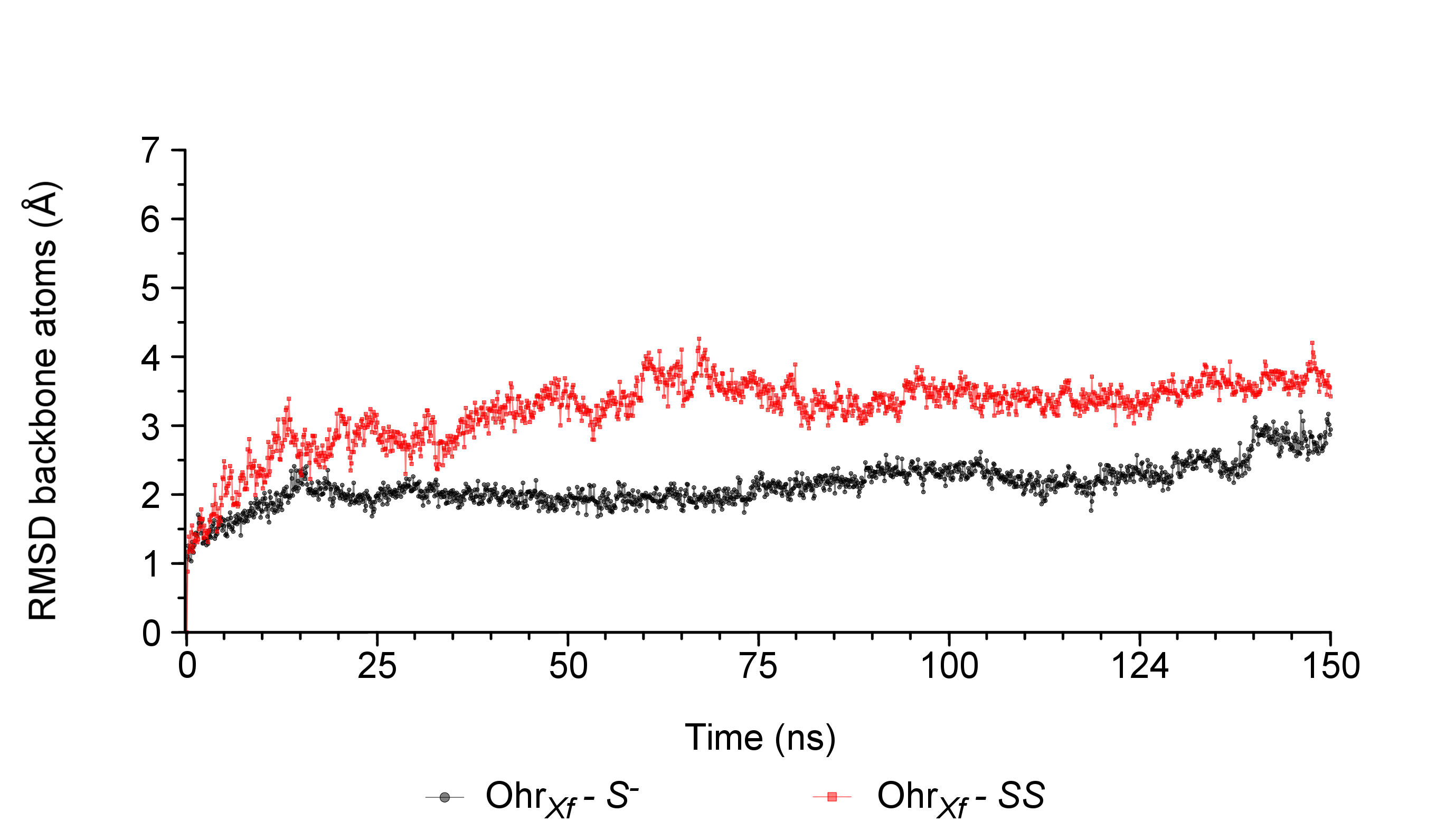

Supplement: S4 Fig — (TIFF) [file pone.0196918.s004.tiff]

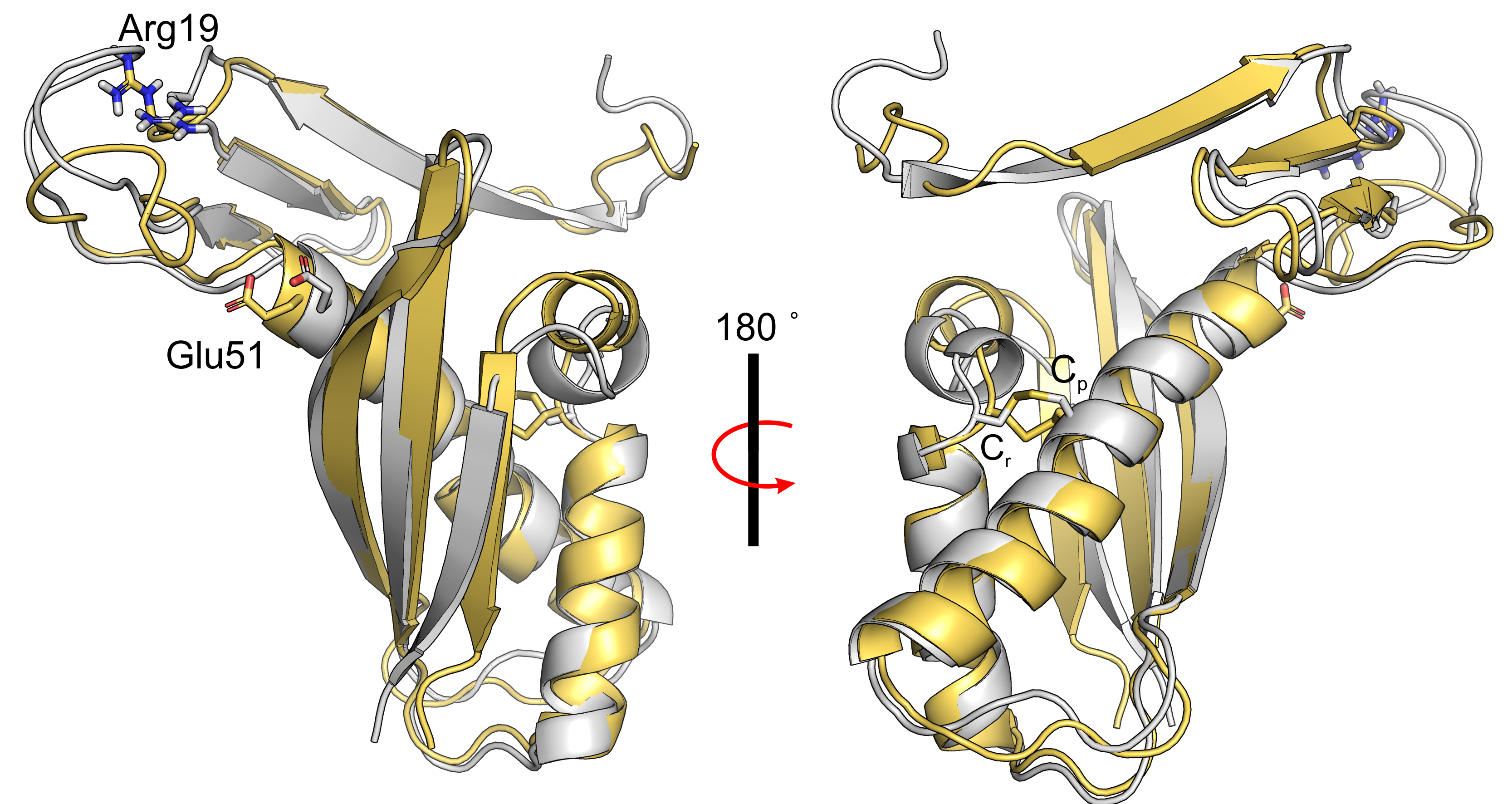

Supplement: S5 Fig — Chain A (gray) superimposed to chain B (yellow) of the XfOhr-SS representative structure, showing a RMSD value of 1.24 Å. (TIFF) [file pone.0196918.s005.tiff]

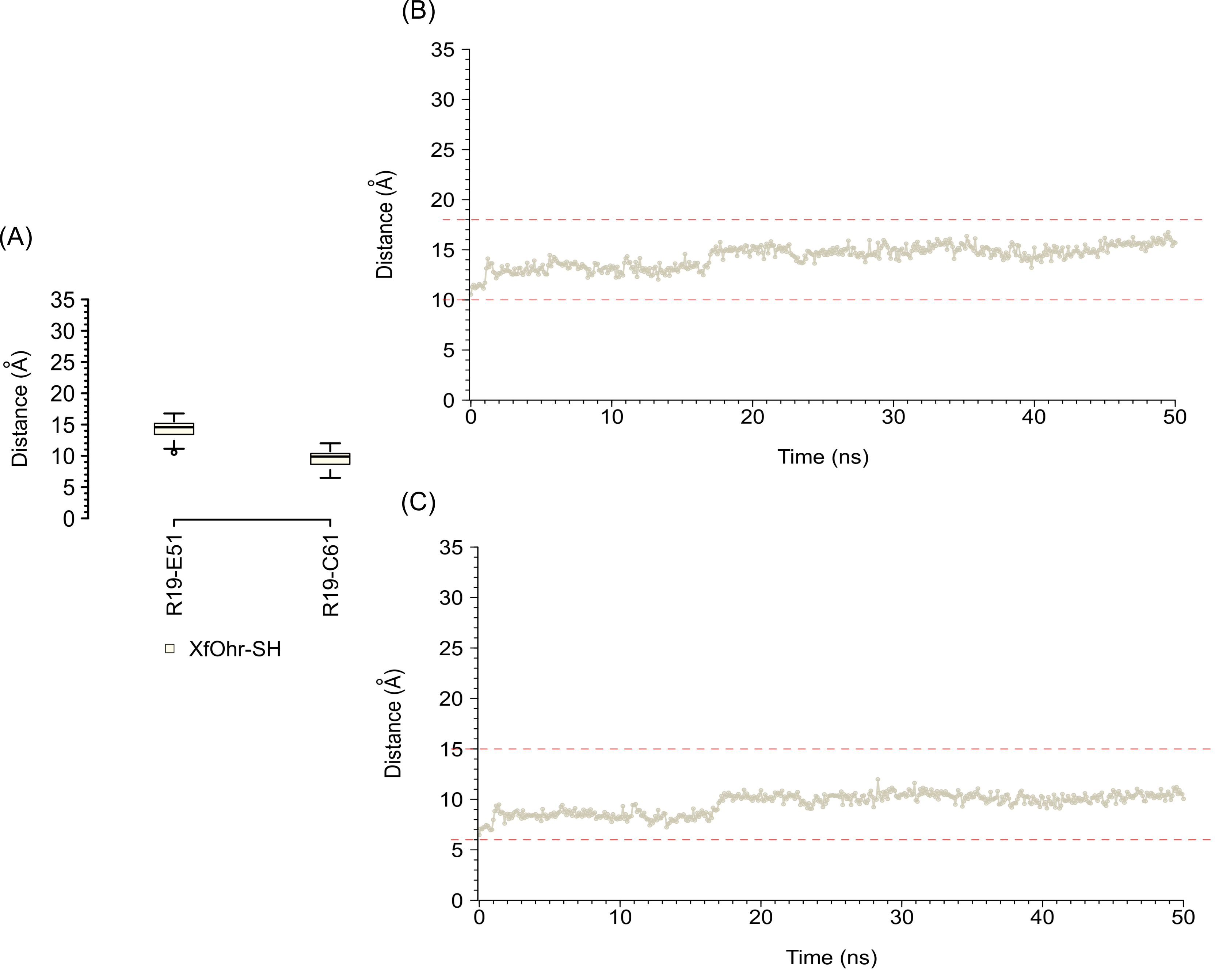

Supplement: S6 Fig — (TIFF) [file pone.0196918.s006.tiff]

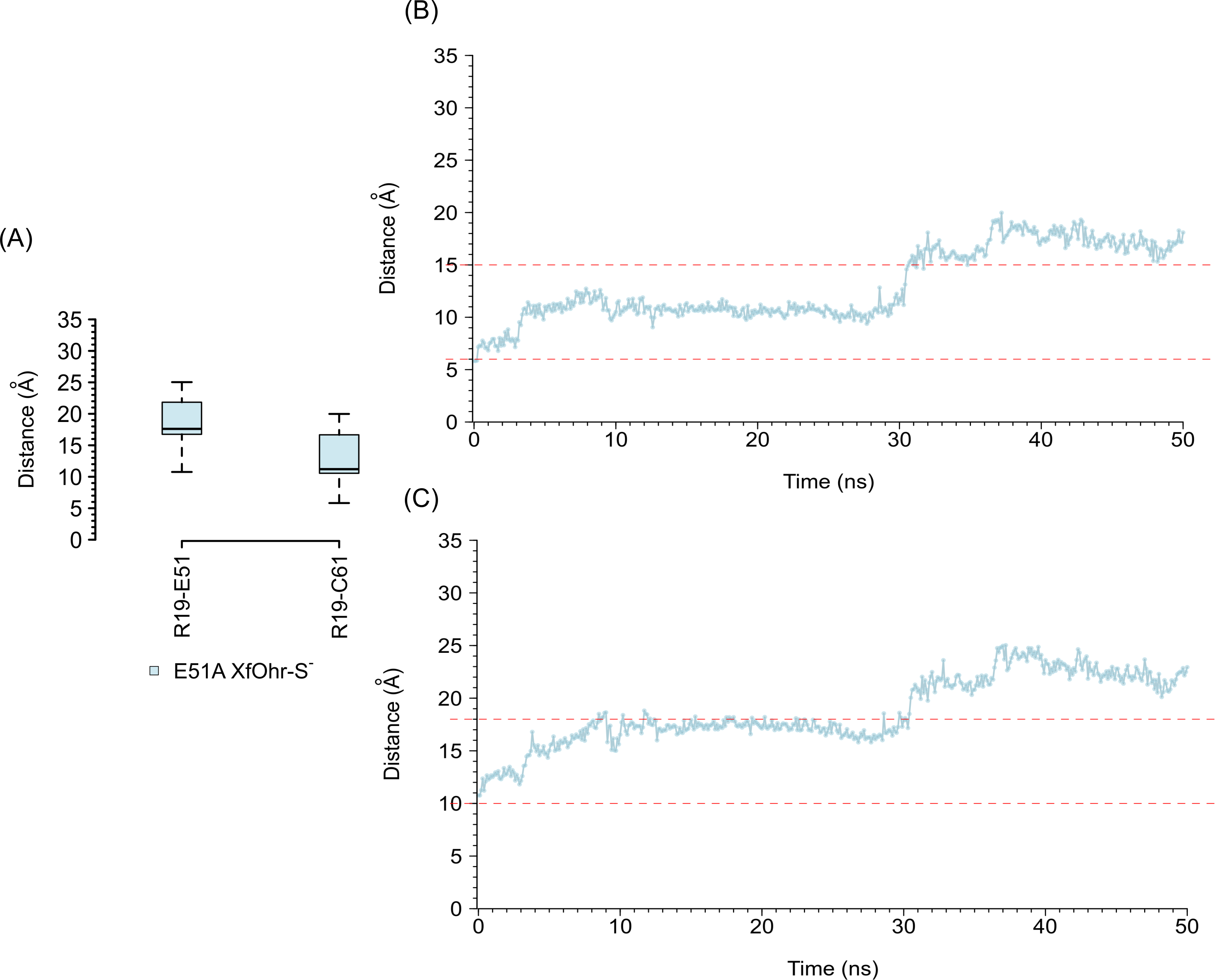

Supplement: S7 Fig — (TIFF) [file pone.0196918.s007.tiff]

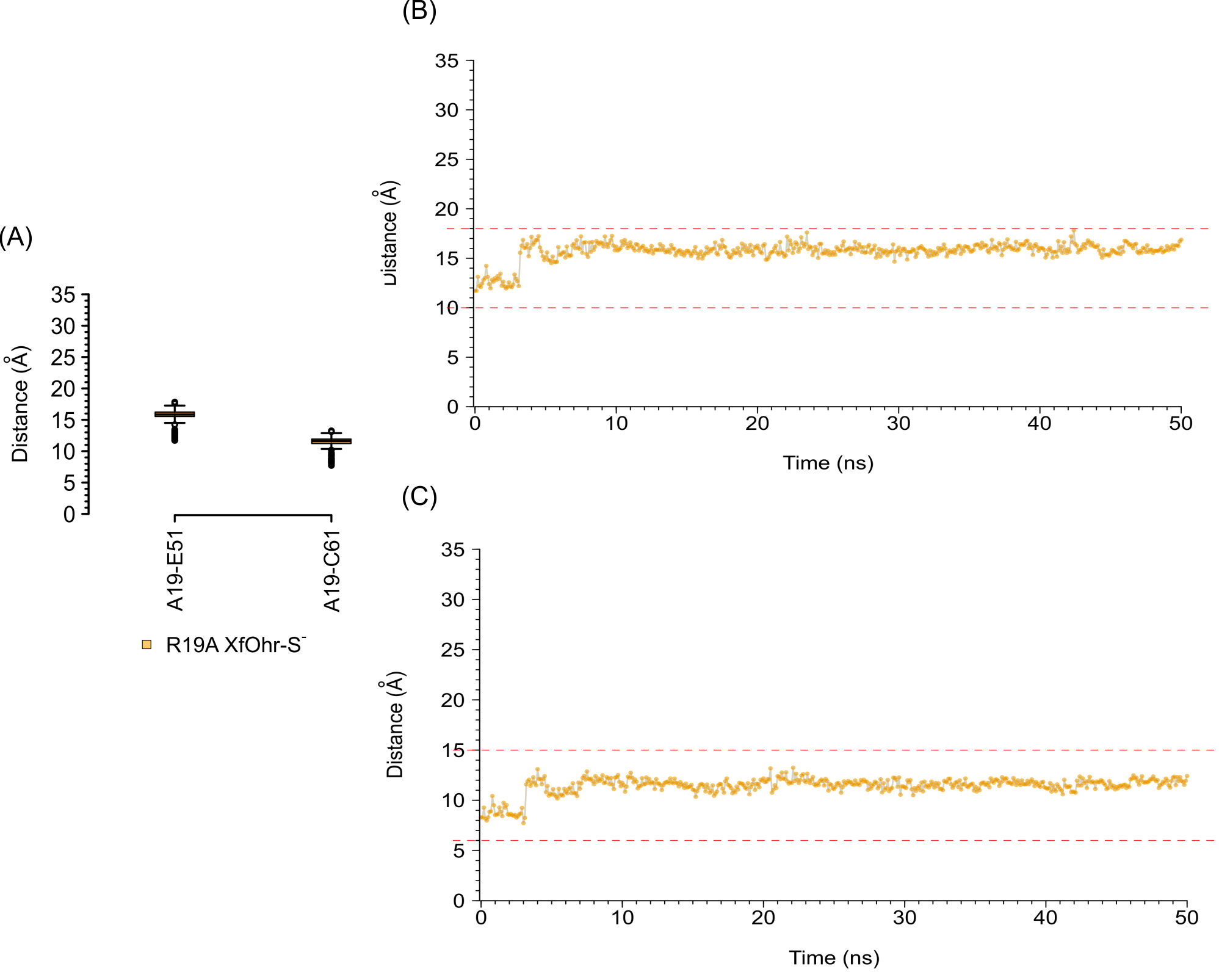

Supplement: S8 Fig — (TIFF) [file pone.0196918.s008.tiff]

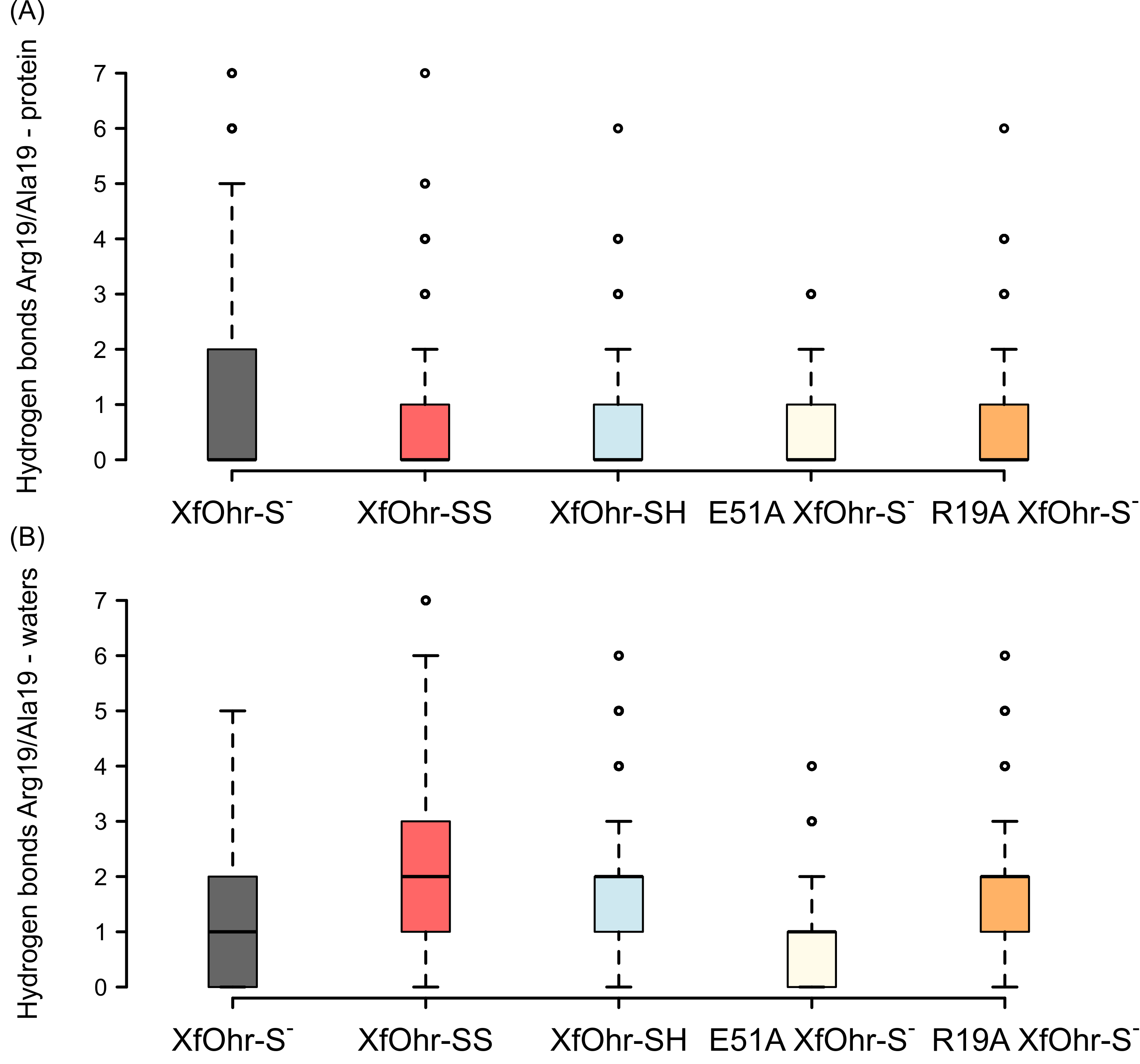

Supplement: S9 Fig — (TIFF) [file pone.0196918.s009.tiff]

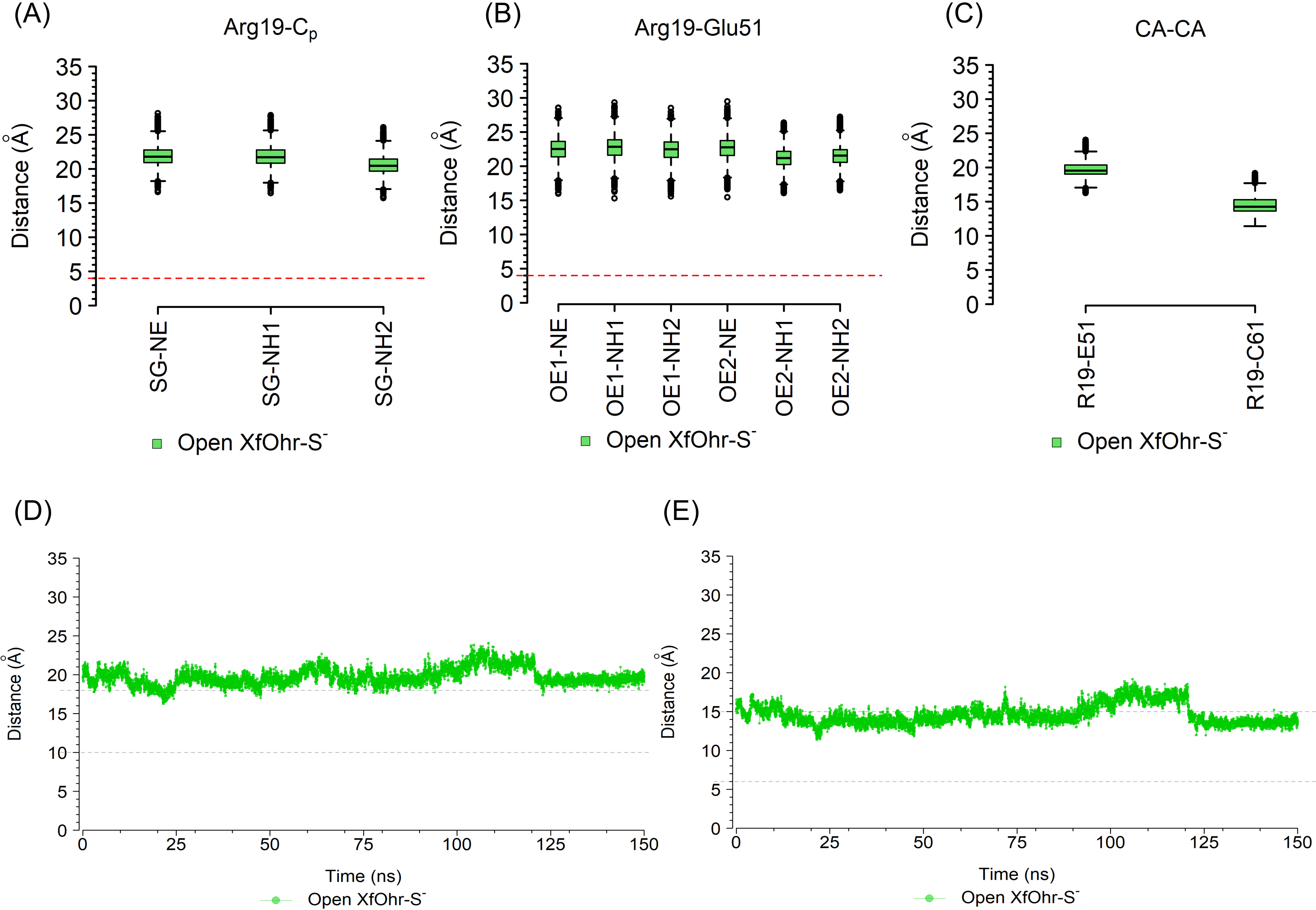

Supplement: S10 Fig — (TIFF) [file pone.0196918.s010.tiff]

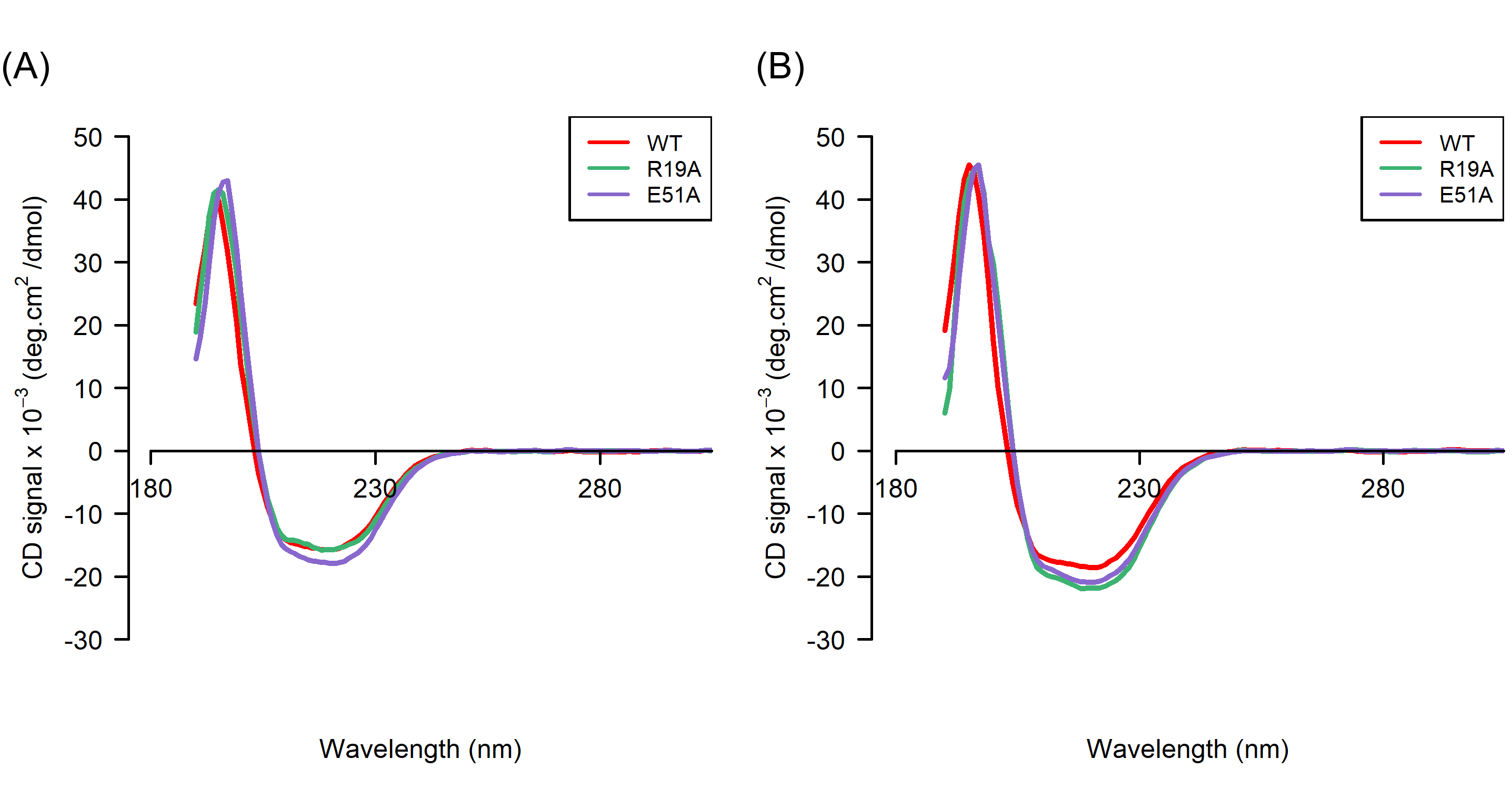

Supplement: S11 Fig — S1 to S8 Videos. (TIFF) [file pone.0196918.s011.tiff]
